# PerFSeeB: designing long high-weight single spaced seeds for full sensitivity alignment with a given number of mismatches

**DOI:** 10.1186/s12859-023-05517-4

**Published:** 2023-10-24

**Authors:** Valeriy Titarenko, Sofya Titarenko

**Affiliations:** 1 School of Biological Sciences, University of Manchester, Oxford Road, Manchester, M13 9PL UK; 2 School of Mathematics, University of Leeds, Woodhouse, Leeds, LS2 9JT UK

**Keywords:** Spaced seeds, Lossless seed, Full sensitivity, Sequence alignment, Mismatch, Indexing

## Abstract

**Background:**

Technical progress in computational hardware allows researchers to use new approaches for sequence alignment problems. For a given sequence, we usually use smaller subsequences (anchors) to find possible candidate positions within a reference sequence. We may create pairs (“position”, “subsequence”) for the reference sequence and keep all such records without compression, even on a budget computer. As sequences for new and reference genomes differ, the goal is to find anchors, so we tolerate differences and keep the number of candidate positions with the same anchors to a minimum. Spaced seeds (masks ignoring symbols at specific locations) are a way to approach the task. An ideal (full sensitivity) spaced seed should enable us to find all such positions subject to a given maximum number of mismatches permitted.

**Results:**

Several algorithms to assist seed generation are presented. The first one finds all permitted spaced seeds iteratively. We observe specific patterns for the seeds of the highest weight. There are often periodic seeds with a simple relation between block size, length of the seed and read. The second algorithm produces blocks for periodic seeds for blocks of up to 50 symbols and up to nine mismatches. The third algorithm uses those lists to find spaced seeds for reads of an arbitrary length. Finally, we apply seeds to a real dataset and compare results for other popular seeds.

**Conclusions:**

PerFSeeB approach helps to significantly reduce the number of reads’ possible alignment positions for a known number of mismatches. Lists of long, high-weight spaced seeds are available in Additional file 1. The seeds are best in weight compared to seeds from other papers and can usually be applied to shorter reads. Codes for all algorithms and periodic blocks can be found at https://github.com/vtman/PerFSeeB.

## Background

Scientists use sequence analysis to understand organisms’ features, structure, and function. By comparing sequences obtained for well-known and unexplored plants or animals, we may understand the biology of the investigated organisms. Currently, experimental techniques cannot provide us with whole sequences but only with many subsequences that we need to concatenate/merge in some way to form a long sequence. Thus, researchers have to solve a general sequence alignment problem before producing any meaningful conclusions related to the biological properties of an organism.

Suppose two long sequences are similar in some way. One sequence (a *reference* sequence) is known, and a researcher wants to find the other sequence. For example, as a reference sequence, we may use a human genome sequence (an “averaged” sequence based on genetic information of several individuals). We want to know a genome sequence for another person, i.e. a patient with a specific disease. The current hardware for genome sequence allows us to find only chunks of the unknown genome of the “patient”. Those chunks (called *reads*) are usually relatively short (hundreds of base pairs) and may contain errors. We assume that the reference and “patient” sequences are similar, so we may use the reference sequence to align a set of reads accounting for possible mismatches.

The standard procedure is to consider each read and find its possible positions within the reference sequence such that the distance between the read and a part of the reference sequence is minimal. Subject to final goals, several definitions of distance as a measure of similarity between two sequences are possible. The distance may depend on all elements of sequences or only elements at specific locations, and it may also involve various transformations of sequences. The similarity between two strings was measured initially using dynamic programming algorithms, see [1–3]. However, due to time complexity, the use of these algorithms became impractical for the increased size of data available. While it is natural to position a read in a way to achieve the smallest distance, the found location may only sometimes be the best one.

With progress related to sequencing hardware and the amount of data provided, we may often simplify the original problem since many reads overlap. So for each position within a reference sequence, tens/hundreds of reads have regions similar to a chosen reference chunk. Ideally, we should align all reads so that there are fewer discrepancies between each other when aligned. Therefore for the initial step of aligning, for each read, we can measure the minimum distance (maximum similarity score) between the read and all possible positions within the reference sequence. Next, however, we should create a list of locations with slightly lower similarity scores. And for the next step, we need to account for all reads to achieve maximum similarity between reads sharing the same regions.

Choosing the best sequence alignment algorithm may also depend on an experimental technique used to acquire data. And data collection procedures tend to be prone to specific errors. For example, scientists usually fragment nucleic acid chains by physical, enzymatic and chemical approaches. However, the enzymatic process produces more insertions/deletions (*indels*) compared to physical techniques [4]. While everyone wants to acquire many high-quality long reads as quickly as possible and for a small cost, there is always a compromise: a large number of short (100–300 bp) reads with a reduced number of errors or a smaller number of long reads ($>1$k bp) with extra errors [5]. Despite different possible approaches to defining distances between two sequences or similarity scores, we should expect that two close or similar sequences should have many identical elements ordered in the same way. Therefore we want to avoid checking all positions within a reference sequence, e.g. billions of positions for a human genome, but consider a limited number of candidate positions where a read and a reference sequence have common subsequences (anchors). We may be tempted to consider only those positions that provide us with the least distance from the read. However, more candidate positions with higher distance values may give us higher similarity scores when dynamic programming algorithms are applied. Therefore we need an algorithm to find possible candidate positions within a specific distance from a read.

In the late 90s, several new algorithms, e.g. BLAST [6, 7], appeared based on ideas of filtration and indexing. Researchers used short sequence fragments. They require pieces from the read to be present in a reference sequence to align a read. The search is sped up using various indexing, which may provide a researcher with “false-matching” positions. The fragments were considered contiguous segments. Once candidate positions based on the complete matching of contiguous regions are found, algorithms extend areas around the common parts to calculate a similarity score (seed-and-extend approach).

Originally only contiguous fragments were considered. However, in [8, 9], *spaced seeds* were introduced when elements of sequences were taken into account at specific positions only. We ignore possible different elements at other positions. For example, one popular spaced seed is 111010010100110111 introduced in [8]. Elements 1 mean that we take into account possible differences, while in the case of elements 0, we ignore them. The total number of 1 -elements is called the *weight* of a seed, and the total number of all elements of a seed is the *length*. For the above seed, the weight is 11 and length 18. Let us be given two sequences ATCATATCCGTAGCCTCT and ATCATAGCCGTTGCATCT of length 18. There are three mismatches (the seventh, twelfth and fifteenth elements from the left), so the sequences are different. However, when the above mask (seed) is applied, only eleven elements are compared, and all these elements are identical. Let the first string belong to a reference sequence and the second string belong to a read. Then the seed provides us with the same “signatures” for these strings. So if we have indexed all substrings in the reference sequence in the same way, we pre-align the read and perform an in-depth comparison after. However, if there are two strings ATCATATCCGTAGCCTCT and ACCGTATCGGTAACCTCT, then we have four mismatches (the second, fourth, ninth and thirteenth elements) and only of them are ignored. Therefore using the indexed library does not allow us to pre-align the read. The goal is to have such seeds that the corresponding indexed library is good for finding similar strings (allowing some number of mismatches) but, at the same time, does not have an excessive number of candidate locations to be checked later.

Some spaced seeds for different weights can be found in [10]. The idea of spaced seeds was extended for other problems: vector [11], indel [12], and neighbour [13] seeds. In ZOOM software [14], spaced seeds are generated to perform alignment with at most two mismatches. In PerM software [15], the authors used so-called *periodic spaced seeds* to improve mapping efficiency. Fast alignment-free string comparison based on spaced seeds (spaced-words) is discussed in [16]. Later approaches based on multiple seeds were used, e.g. [17, 18] (a good review can be found in [19]). Seeds in a multiple-seed environment are designed to have less overlap and thus increase the chances of hitting common regions. A good bibliography related to spaced seeds can be found in [20]. While papers for the original BLAST [6] and PSI-BLAST [7] algorithms have very high number of citations (more than 70 000 and 60 000), the developers of the modern versions of BLAST agree that the productivity of using spaced seeds is much higher compared to contiguous seeds [21]. SpeedBLAST is an example of an extension of original BLAST software based on spaced seeds which is superior in terms of efficiency, especially in the case of twilight zone hits [22]. Inspired by the results shown in [22] the authors consider the next step to be the modification of the BLAST algorithm to take advantage of the spaced seeds designed in this work. The full design of the algorithm for the local alignment is out of the scope of the current study.

Seed design may also deal with a non-binary alphabet. For example, in YASS [23] a three-letter alphabet (#, @, -) is used, where # stands for a nucleotide match, @ is for a match or transition ($\texttt{A}\leftrightarrow \texttt{G}$ or $\texttt{C}\leftrightarrow \texttt{T}$ mutations) and - is used when we ignore corresponding symbols.

Computational resources available to researchers for the past 20 years have also improved significantly. For example, even for a budget computer it is possible to create a library of records, i.e. data structures of pairs (“key”, “value”). A human genome has a length of ${\approx } 3.2\times 10^9 < 2^{32}$ bp. Therefore each position (“key”) within the sequence can be represented as a 32-bit number. Suppose the corresponding “value” for a given position depends on *n* elements around the position in a predefined order (*n* is the weight of a seed). If we avoid undefined areas, then each element is one of the following four symbols $\texttt{A}$, $\texttt{C}$, $\texttt{G}$, $\texttt{T}$, i.e. requires only 2 bits for storage, e.g. $\mathtt{A=00}$, $\mathtt{C=01}$, $\mathtt{G=10}$ and $\mathtt{T=11}$. Therefore each record (“key”, “value”) may require $(32 + 2n)$ bits. Firstly, we create a library of records and all these records are ordered by “key” elements which are unique for each record. Secondly, we sort all records by “values”. The library sorted in this way may have multiple records for the same index number (“value”). The total size of the library is at most $3.2\cdot 10^9\cdot (32+2n)$ bits or $8\cdot 10^8\cdot (16+n)$ bytes. It is also possible to split the data into smaller chunks. For example, each chunk has the same first 16 bits of “values”, and we have $2^{16}=65536$ chunks but shorter $(16+2n)$-bit records or $8\cdot 10^8\cdot (8+n)$ bytes in total. So, for $n=16$, the storage requirement is 17.9 GB, for $n=32$, 48 and 64 we get 29.8, 41.7 and 53.6 GB, respectively.

The human genome contains repeated regions, so there may be extreme cases of many “keys” or no “keys” for specific “values”. However, by increasing the weight of a seed by one, we reduce the number of “keys” (positions) for each “value” four times. So, having high-weight seeds is our preference. On the other hand, the “patient” genome varies from the reference genome we know. Therefore the corresponding reads for the “patient” genome may also have mismatches, insertions, and deletions. Consequently, a candidate seed should cope with the presence of such deviations. In this paper, we only consider spaced seeds to deal with a known maximum number of mismatches (single-nucleotide polymorphisms or SNPs).

The sensitivity of seeds is usually measured using probabilistic approaches. In this paper, we consider an extreme case of *full sensitivity* seeds under the assumption that a read has at most $n_m$ mismatches. This means that these seeds should allow us to find *all* candidate positions. Full sensitivity seeds are also known as *lossless seeds* [24] and were applied to filtration problems. A lossless filtration means that all fragments will be found (in the case of *lossy* filtration, we may miss some of them).

There may be several seeds of the maximum weight. Longer seeds require us to generate and check fewer index values in the library of records. We observed that most long high-weight seeds have a periodic structure. However, this structure is not a perfect one like discussed in [24] where a seed is made of several repetitions of structure A separated by structure B. We have an integer number of repetitions of structure A and a “remainder” of this structure (several first elements of structure A). The size of a periodic block has a simple relation with the lengths of reads and the seed. It is possible to generate those periodic blocks within a reasonable time frame (from a fraction of a second for blocks less than 30 symbols to several hours for cases of 50 symbols).

In the Methods section, we first discuss how to align reads to a reference sequence when an indexed library of records is used in the presence of possible mismatches. Then, we touch on ideas of software implementations affecting computational performance and how they dictate the choice of seeds. Methods to validate candidate seeds and approaches to speed up validation are proposed. Having a list of all valid short seeds, we attempt to design longer seeds and explain methods for seed extension. There may be too many valid seeds, and it is hard to construct them all using the seed extension approach. Therefore for long reads, it is better to create so-called periodic seeds made of several same concatenated blocks and a “remainder”. Rules to form these blocks, as well as whole seeds to be valid, are discussed. In the Results section, we summarise the properties of periodic seeds, list the most popular spaced seeds and compare them with seeds designed with the PerFSeeB approach. Software tools are available to create library of records for given seeds and find candidate positions for reads. By applying the PerFSeeB approach to a real dataset, we show that one should check significantly fewer candidate positions for several possible mismatches.

## Methods

### Sequence alignment

Let there be an alphabet ${\mathcal{A}}$ of symbols and two strings *x* and *y* of length *n* consisting of characters from ${\mathcal{A}}$. The term distance *d*(*x*, *y*) between two strings *x* and *y* was introduced in [25] for binary alphabet ${\mathcal{A}} =\{0, 1\}$ in the space of $2^n$ points as the number of mismatches between points *x* and *y*. In general, we may introduce a list of elementary operations which convert a source string *x* into a target string *y*. Each function may have a different cost. The distance is then a sum of all costs. If we cannot transform *x* into *y*, then the cost is $\infty$. A good review of possible distances can be found in [26]. The most common distances are the following ones.

1. Levenshtein (or edit) distance (insertions, deletions, substitutions are allowed at equal cost of 1) [27]. For example, we may transform word “health” into “shale” as $\text{``health''}\rightarrow \text{``healt''}\rightarrow \text{``heale''}\rightarrow \text{``sheale''}\rightarrow \text{``shale''}$ (2 deletions, 1 substitution, 1 insertion), so $d(\text{``health''},\text{``shale''}) = 4$.
2. Hamming distance (only substitutions) [28].
3. Longest common subsequence distance (insertions/deletions) [29].

Suppose we have two sequences $x=\texttt{CTTGTCGTTGGAGATCGGAAGAGCA}$ and $y=\texttt{TAGGTGCTCG}$ of length $n_1=25$ and $n_2 = 10$, respectively. We define $m=n_1 - n_2 + 1 = 16$ and for each index $j=1,\ldots ,m$ we find distance $d_j=\sum _{k=1}^{n_2} \delta (x_{j+k}, y_k)$, where $\delta (a,b) = 1$ if symbols *a* and *b* are different, otherwise $\delta (a,b)=0$. For example, to find $d_8$ we align the corresponding strings and calculate values for $\delta (x_{j+k}, y_k)$:

$$\begin{aligned} \begin{array}{ll} \text{string 1} &{}\texttt{CTTGTCGTTGGAGATCGGAAGAGCA}\\ \text{string 2} &{}\texttt{\_\_\_\_\_\_\_TAGGTGCTCG\_\_\_\_\_\_\_\_}\\ \delta _k &{}\texttt{\_\_\_\_\_\_\_0100101000\_\_\_\_\_\_\_\_} \end{array} \end{aligned}$$

So, $d_8 = 3$, in a similar way we get $d = (6, 5, 9, 7, 6, 9, 9, 3, 7, 8, 7, 8, 8, 8, 7, 7)$. We compare all $n_2$ symbols for both sequences. However, by introducing spaced seeds we may ignore some symbols. For example, we may use seed of length $n_2$: 1011011111 (binary notation used in [8]) or #-##-##### (notation used in [9]), where 1 or # stand for a match and 0 or - for ‘do not care’. Thus, to find the distance $d_8$ accounting for the spaced seed we get

$$\begin{aligned} \begin{array}{ll} \text{string 1} &{}\texttt{CTTGTCGTTGGAGATCGGAAGAGCA}\\ \text{string 2} &{}\texttt{\_\_\_\_\_\_\_TAGGTGCTCG\_\_\_\_\_\_\_\_}\\ \delta _k&{}\texttt{\_\_\_\_\_\_\_0100101000\_\_\_\_\_\_\_\_}\\ \text{seed}&{}\texttt{\_\_\_\_\_\_\_\#}\text{-}\texttt{\#\#}\text{-}\texttt{\#\#\#\#\#\_\_\_\_\_\_\_\_}\\ \text{new}\, \delta _k &{}\texttt{\_\_\_\_\_\_\_0000001000\_\_\_\_\_\_\_\_} \end{array} \end{aligned}$$

or $d_8 = 1$. Similarly, we get $d = (5, 3, 7, 6, 5, 7, 7, 1, 5, 6, 7, 6, 7, 6, 5, 5)$. From now, we will use binary notation as it is closer to binary numbers utilized for storage and computation.

Now let us consider the library of records. Suppose we have a contiguous seed $s=\texttt{1111}$ of weight $w=4$. So, if we take the first four symbols of vector *x*, then we encode string $\texttt{CTTG}$ as $1 + 4\cdot 3 + 4^2\cdot 3 + 4^3\cdot 2 = 1 + 12 + 48 + 128 = 189$ (if we set $\texttt{A} = 0$, $\texttt{C} = 1$, $\texttt{G} = 2$, $\texttt{T} = 3$). Therefore the library of records for the reference sequence *x* and seed *s* has 22 pairs (“key”, “value”):

$$\begin{aligned} \begin{array}{llll} (1, \texttt{CTTG} = 189) &{} (2, \texttt{TTGT} = 239) &{} (3, \texttt{TGTC} = 123) &{} (4, \texttt{GTCG} = 158) \\ (5, \texttt{TCGT} = 231) &{} (6, \texttt{CGTT} = 249) &{} (7, \texttt{GTTG} = 190) &{} (8, \texttt{TTGG} = 175) \\ (9, \texttt{TGGA} = 43) &{} (10, \texttt{GGAG} = 138) &{} (11, \texttt{GAGA} = 34) &{} (12, \texttt{AGAT} = 200) \\ (13, \texttt{GATC} = 114) &{} (14, \texttt{ATCG} = 156) &{} (15, \texttt{TCGG} = 167) &{} (16, \texttt{CGGA} = 41) \\ (17, \texttt{GGAA} = 10) &{} (18, \texttt{GAAG} = 130) &{} (19, \texttt{AAGA} = 32) &{} (20, \texttt{AGAG} = 136) \\ (21, \texttt{GAGC} = 98) &{} (22, \texttt{AGCA} = 24) &{} \end{array} \end{aligned}$$

Similarly, we may find “values” for sequence *y*:

$$\begin{aligned} \texttt{TAGG}&= 163, \texttt{AGGT} = 232, \texttt{GGTG} = 186, \texttt{GTGC} = 110,\\ \texttt{TGCT}&= 219, \texttt{GCTC}= 118, \texttt{CTCG} = 157. \end{aligned}$$

We may see that no “values” for *y* can be found in the library generated for *x*. Therefore the use of “seed and extend” approach does not allow us to align *y* with respect to *x*, since no candidate positions can be found.

Suppose we use seed $s=\texttt{101011}$ (also of weight 4). We generate a new library for *x* (now containing 20 pairs):

$$\begin{aligned} \begin{array}{llll} (1, \texttt{CTTC} = 125) &{} (2, \texttt{TGCG} = 155) &{} (3, \texttt{TTGT} = 239) &{} (4, \texttt{GCTT} = 246) \\ (5, \texttt{TGTG} = 187) &{} (6, \texttt{CTGG} = 173) &{} (7, \texttt{GTGA} = 46) &{} (8, \texttt{TGAG} = 139) \\ (9, \texttt{TGGA} = 43) &{} (10, \texttt{GAAT} = 194) &{} (11, \texttt{GGTC} = 122) &{} (12, \texttt{AACG} = 144) \\ (13, \texttt{GTGG} = 174) &{} (14, \texttt{ACGA} = 36) &{} (15, \texttt{TGAA} = 11) &{} (16, \texttt{CGAG} = 137) \\ (17, \texttt{GAGA} = 34) &{} (18, \texttt{GAAG} = 130) &{} (19, \texttt{AGGC} = 104) &{} (20, \texttt{AACA} = 16) \end{array} \end{aligned}$$

For sequence *y* we get “values”:

$$\begin{aligned} \texttt{TGTG} = 187, \texttt{AGGC} = 104, \texttt{GTCT} = 222, \texttt{GGTC} = 122, \texttt{TCCG} = 151. \end{aligned}$$

There are three “values” (187, 104 and 122) found in the new library. Therefore we need to try three positions: $5 - 1 + 1 = 5$, $19 - 2 + 1 = 18$, $11- 4+1 = 8$. For the first position (5) we get

$$\begin{aligned} \begin{array}{ll} \text{string 1} &{}\texttt{CTTGTCGTTGGAGATCGGAAGAGCA}\\ \text{string 2} &{}\texttt{\_\_\_\_TAGGTGCTCG\_\_\_\_\_\_\_\_\_\_\_}\\ \delta _k &{}\texttt{\_\_\_\_0101001111\_\_\_\_\_\_\_\_\_\_\_} \end{array} \end{aligned}$$

and $d_5 = 6$, the second position (18) cannot be used, since the aligned string *y* will be out of the range for string *x* and the third position (8) gives us

$$\begin{aligned} \begin{array}{ll} \text{string 1} &{}\texttt{CTTGTCGTTGGAGATCGGAAGAGCA}\\ \text{string 2} &{}\texttt{\_\_\_\_\_\_\_TAGGTGCTCG\_\_\_\_\_\_\_\_}\\ \delta _k &{}\texttt{\_\_\_\_\_\_\_0100101000\_\_\_\_\_\_\_\_} \end{array} \end{aligned}$$

and $d_8 = 3$. So, the seed $\texttt{101011}$ allows us to find the best alignment position of *y* with respect to *x*.

We are interested in aligning genetic sequences. Reference sequences may consist of several separate sequences. For example, T2T_CHM13v2.0 Telomere-to-Telomere assembly (see [30]) contains 24 separate sequences of four symbols A, C, G, T. However, GRCh38.p14 release has 705 separate sequences (chromosomes, genomic scaffolds and patches) and five symbols (A, C, G, T, and N for unknown symbols). We may always concatenate separate sequences into one long sequence by adding extra “void” symbols in between to avoid forming library records containing symbols from different original sequences. In the case of the T2T reference genome, we may use two bits to encode each symbol. For the GRCh38.p14 reference genome, we have five possible values to combine every three consecutive symbols into a 7-bit number (as $5^3 = 125 < 128 = 2^7$). So, if we do not use any data compression, then storing the T2T sequence may require 0.73 GB, and for GRCh38.p14, we need about 0.84 GB. These numbers are minimal, even for a budget computer. Therefore we may use more storage space to achieve better performance. The 2-bit encoding is preferable even if we need to exclude some substrings containing any character except A, C, G, T.

Modern computers allow users to exploit SIMD (single instruction, multiple data) properties of CPUs (central processing units). For example, one instruction can be applied to a 128-, 256- or 512-bit number. CPUs can do various logical and shift operations very fast. A list of Intel’s SIMD intrinsics designed for various CPU instruction sets can be found in [31]. Most computers (servers, workstations and home computers) built for the past decade support the 128-bit instruction set. Thus, we will use 128-bit instructions. There are similar instructions for 256- and 512-bit numbers, so the ideas discussed in the paper can also be applied for new architectures.

We may split the reference sequence into groups of 32 symbols, then form 128 bits such that the first 32 bits are 1s if the corresponding symbols are A, the second 32 bits are for symbols C, the third and fourth groups of 32 bits are for G and T, respectively. For example, the string CATAGNCACGTGATCCTAGNCATGTTACCTGT of 32 symbols has the following components

$$\begin{aligned} \begin{array}{cll} \text{sequence} &{} \texttt{CATAGNCACGTGATCCTAGNCATGTTACCTGT} &{} \text{32-bit number}\\ \texttt{A} &{} \texttt{01010001000010000100010000100000} &{} \texttt{0x0422108a}\\ \texttt{C} &{} \texttt{10000010100000110000100000011000} &{} \texttt{0x1810c141}\\ \texttt{G} &{} \texttt{00001000010100000010000100000010} &{} \texttt{0x40840a10}\\ \texttt{T} &{} \texttt{00100000001001001000001011000101} &{} \texttt{0xa3412404}\\ \texttt{A|C|G|T} &{} \texttt{11111011111111111110111111111111} &{} \texttt{0xfff7ffdf} \end{array} \end{aligned}$$

If an element is N symbol, then the corresponding bits of all four 32-bit numbers are zeros. We use symbol “|” for logical OR operation, similarly, symbol “&” is for logical AND operation.

Let there be two sequences of length 32. We want to find how many symbols are the same for them (if one symbol is N, then there is no match).

$$\begin{aligned} \begin{array}{cc} \texttt{m1} &{} \texttt{CATAGNCACGTGATCCTAGNCATGTTACCTGT}\\ \texttt{m2} &{} \texttt{GCCTCAGTTTTCACTCTATCAATATGTAATAA}\\ \texttt{m1 \& m2} &{} \texttt{\_\_\_\_\_\_\_\_\_\_T\_A\_\_CTA\_\_\_AT\_T\_\_\_\_T\_\_} \end{array} \end{aligned}$$

There are nine such symbols. We may apply logical AND operation for corresponding 32-bit components of m1 and m2

$$\begin{aligned} \begin{array}{ccccc} &{} \texttt{A} &{} \texttt{C} &{} \texttt{G} &{} \texttt{T}\\ \texttt{m1} &{} \texttt{0x0422108a} &{} \texttt{0x1810c141} &{} \texttt{0x40840a10} &{} \texttt{0xa3412404}\\ \texttt{m2} &{} \texttt{0xd8b21020} &{} \texttt{0x0008a816} &{} \texttt{0x02000041} &{}\texttt{0x25454788}\\ \texttt{m1 \& m2} &{} \texttt{0x00221000} &{} \texttt{0x00008000} &{} \texttt{0x00000000} &{} \texttt{0x21410400} \end{array} \end{aligned}$$

Since 0x00221000 | 0x00008000 | 0x00000000 | 0x21410400 = 0x21639400, then the number of 1-bits in 0x21639400 equals 9 and is the total number of matches. Now we approach the main goal of the manuscript, i.e. how to design seeds that will allow us to find candidate positions.

### Choice of seeds

Suppose there is a short sequence we want to align (*read*) for a known long *reference* sequence. The length of the read is $n_r$. We may always assume that a read contains only four symbols (A, C, G and T). While there may be cases of reads containing symbols N for unknown letters, the number of such cases is often negligible. Let there be a seed *s* of length $n_s$ (total number of all bits, 1s and 0s) and weight *w* (the number of 1s). If we have 1-bit, then the corresponding symbol for a given sequence is taken into account; otherwise (in the case of 0-bit), it is ignored. We may also assume that a seed’s first and last bits are 1-bits. *Contiguous seeds* have only 1-bits, and the lengths and weights of these seeds are equal. *Spaced seeds* have at least one 0-bit.

Seeds allow us to form a shorter sequence from a longer one. We apply a seed of length $n_s$ to a sequence of the same length and form a new sequence of length *w* when symbols of the original sequence are in front of 1-bits of the seed. We consider the new shorter sequence as a characteristic/signature of the longer sequence. In the case of a 4-letter alphabet, we may perform one-to-one mapping of the short sequence and a 2*w*-bit number. We use these numbers to find pairs of (“key”, “value”) in the library of records generated for the reference sequence. These pairs should have “value”-components equal to our 2*w*-bit numbers. We may generate only $(n_r-n_s+1)$ 2*w*-bit numbers for each read since the first and last bits of a seed should be within the read’s boundaries.

Suppose we have a seed of weight four, and by applying it at some reads’ position, we get “value” ACGT. Let there be *N* pairs in the reference library with the same “value”. Now we expand the original seed and assume the new seed at the chosen position will also be within reads’ boundaries. This means that the new seed may provide us with four new “values” (ACGTA, ACGTC, ACGTG, ACGTT). The number of (“key”, “value”) pairs found for the new seeds will be less or equal to *N*. For example, if the reference sequence is AGTGACGT, then we have one pair for ACGT and no pairs for the 5-symbol sequences. Of course, it might happen that the number of pairs in the library generated for “value” ACGTA is *N*, and there are no pairs found for ACGTC, ACGTG and ACGTT. However, we may often think that the number of pairs for each 5-symbol sequence is around *N*/4.

For a library of records generated for a reference sequence we may estimate the total number of pairs found for a given “value” as $\alpha /4^w$, where $\alpha$ is a constant. We generate $(n_r-n_s+1)$ numbers (“values”) for a read. Based on the above empirical rule, we should expect to consider about $\alpha (n_r-n_s+1)/4^w$ candidate positions in the reference sequence where we try to align the given read. Each “value” is found for a substring of a read with a given shift. Since the goal is to pre-align the read, the “keys” we found should be corrected by the corresponding shifts of a substring to the read’s first element. For example, if a read has an exact match within a read, then all its “values” should also have an exact match. So, in the best scenario, we should have about $\alpha /4^w$ unique candidate positions (when a read can be placed exactly at several locations of the reference sequence). However, it is better to estimate the number of candidate positions from the above as

1 $$\begin{aligned} \alpha \frac{n_r-n_s+1}{4^w}. \end{aligned}$$

This number indicates what steps we should perform to reduce the number of candidates’ locations and thus improve performance of sequence alignment algorithms. The steps are

1. find seeds of maximum weight,
2. among the found seeds choose seeds of maximum length.

Of course, the trivial approach is to maximise $n_s$ and *w*. So, a contiguous seed of length $n_r$ is the best candidate. However, there are restrictions as we should account for various mismatches between the reference and “patient” sequences. In this paper we consider only pointwise mismatches (SNPs) and suppose all reads have at most $n_m$ mismatches. Hereafter, we assume that all seeds we try to find meet this rule.

- Let there be two arbitrary reference and read sequences such that the read can be aligned with no more than $n_m$ mismatches. Then at least one “value” for the read should be paired with the records from the library generated for the reference sequence.

Suppose there are two seeds $s_1$ and $s_2$ and we may align $s_1$ with respect to $s_2$ in such a way that all 1-bits of seed $s_1$ are also present in $s_2$. Then $s_1$ is a *subset* of $s_2$ and can be denoted as $s_1\subset s_2$. For example, if $s_1=\texttt{10111}$, $s_2=\texttt{11011101}$, then by shifting $s_1$ by one element to the right we get

$$\begin{aligned} \begin{array}{ll} s_2 &{} \texttt{11011101}\\ s_1 &{} \texttt{\_10111\_\_} \end{array} \end{aligned}$$

Note that there may be multiple possible shifts and $s_1$ may have more 0-bits. For example, $s_3=\texttt{10101}\subset s_1$ and we get two possible alignments

$$\begin{aligned} \begin{array}{ll} s_2 &{} \texttt{11011101}\\ s_3 &{}\texttt{\_10101\_\_}\\ s_3 &{} \texttt{\_\_\_10101} \end{array} \end{aligned}$$

Seed $$s_4=\texttt{1101011} \not \subset s_2$$.

The human genome has many repeated regions, so several candidate seeds have similar weights/lengths. Therefore, it is reasonable to choose a seed with a more uniform distribution of 1-bits rather than seeds with grouped 1-bits. In any case, if we have many repeated experiments providing us with reads of the same length for a known reference sequence, it is worth performing a statistical analysis of the “keys” distribution to avoid cases when for some “values” we have thousands of “keys”.

According to [24], we may consider two main alignment problems: *lossless* alignment when we detect *all* locations in the reference sequence and *lossy* alignment when we may miss some of them. As for any statistical analysis, we may have true-positive (TP), true-negative (TN) and false-positive (FP) and false-negative (FN) events. We aim to find seeds that provide us with lossless alignment. Therefore for a given read and seed, we construct all “values” according to the procedure described above, and then we do not miss any candidate location. Thus the number of false negative events is zero. Since

2 $$\begin{aligned} \text{sensitivity} = \frac{\text{TP}}{\text{TP} + \text{FN}}, \end{aligned}$$

then for our seeds $\text{FN} = 0$ and $\text{sensitivity} = 100\%$. So, our seeds have *full sensitivity* or we have *lossless* seeds.

### Seed validation

Let us consider an example. We check if seed $s=\texttt{110101011001011}$ is a full sensitivity seed for reads of length 20 and at most two mismatches. The length of *s* is 15. Therefore there are $(20-15+1) = 6$ possible positions of the seed with respect to a read. We shift the seed and pad it with zeros (five extra zeros for each row). Thus we get the following six rows of length 20:

$$\begin{aligned} L_1 &{} = {} \texttt{11010101100101100000},\\ L_2 &{} = {} \texttt{01101010110010110000},\\ L_3 &{} = {} \texttt{00110101011001011000},\\ L_4 &{} = {} \texttt{00011010101100101100},\\ L_5 &{} = {} \texttt{00001101010110010110},\\ L_6 &{} = {} \texttt{00000110101011001011}. \end{aligned}$$

A seed is *valid* if for any two positions of mismatches within a read, there is at least one row of the matrix with both 0-elements at the given columns. For example, we choose columns 7 and 12, then we may pick up the third row with both 0-elements (underlined):

$$\begin{aligned} \mathtt{001101\underline{0}1011\underline{0}01011000} \end{aligned}$$

Note that 0-elements can be from the original seed or the padded ones. For example, if we choose columns 5 and 16, then the last row of the matrix has both 0-elements (the first 0-element is the padded element):

$$\begin{aligned} \mathtt{0000\underline{0}1101010110\underline{0}1011} \end{aligned}$$

However, if we choose columns 4 and 13, then for each row of the matrix there is at least one 1-element:

$$\begin{aligned} \mathtt{110\underline{1}01011001\underline{0}1100000}\\ \mathtt{011\underline{0}10101100\underline{1}0110000}\\ \mathtt{001\underline{1}01010110\underline{0}1011000}\\ \mathtt{000\underline{1}10101011\underline{0}0101100}\\ \mathtt{000\underline{0}11010101\underline{1}0010110}\\ \mathtt{000\underline{0}01101010\underline{1}1001011} \end{aligned}$$

Therefore seed $s=\texttt{110101011001011}$ is not a valid full sensitivity seed for $n_m=2$, $n_r =20$. To check validity we should check all possible combinations, for the above example we have to check $C_{20}^2\equiv 20!/2!(20-2)! = 190$ cases, where $m!\equiv 1\cdot 2\cdot 3 \cdots m$. For example, we have two sequences AAAAAAAAAAAAAAAAAAAA (reference sequence) and AAATAAAAAAAATAAAAAAA (read). For the reference sequence we create its library of records:

$$\begin{aligned}&(1, \texttt{AAAAAAAAA}),\quad (2, \texttt{AAAAAAAAA}),\quad (3, \texttt{AAAAAAAAA}),\\&(4, \texttt{AAAAAAAAA}),\quad (5, \texttt{AAAAAAAAA}),\quad (6, \texttt{AAAAAAAAA}), \end{aligned}$$

i.e. all “values” are the same. The read provides us with the following six “values”:

$$\begin{aligned}&\texttt{AATAAAAAA},\qquad \texttt{AAAAAATAA},\qquad \texttt{ATAAAAAAA},\\&\texttt{TAAAAAAAA},\qquad \texttt{AAAAATAAA},\qquad \texttt{AAAATAAAA}. \end{aligned}$$

There is no read’s “value” equal to “values” in the library.

Now consider a general case. Let there be a seed *s* of length $n_s$. We want to check if the seed meets the full sensitivity requirement for any read of length $n_r$ and a maximum number of $n_m$ mismatches. Suppose $n_m$ and $n_r$ are set. Then we create $n_m$-vector of indices $i_k$, $k=1,2,\ldots ,n_m$ such that $1\le i_k\le n_r$. By definition, the length of a seed is not greater than the length of a read, $n_s\le n_r$. We may shift the first element of a seed by $\delta$ with respect to the first element of the read. As the last element of the seed should be within the read’s elements, then $\delta$ can vary from 0 to $(n_r-n_s)$. For each value of $\delta$, we check that none of the 1-elements of the seed shifted by $\delta$ has indices $i_k$, $k=1,2,\ldots ,n_m$. If for any possible combination of indices $i_k$, there is at least one value of $\delta$ (depending on $i_k$, $k=1,\ldots ,n_m$), the requirement is met, then the seed is a *valid* seed for given $n_m$ and $n_r$.

Parameter $\delta$ has $(n_r-n_s+1)$ values. By padding the seed vector with 0-elements from left and right, we can form $(n_r-n_s+1)$ vectors of length $n_r$. We pad from left and right and the total number of 0-elements to be used for each vector is $(n_r-n_s)$. One may perform padding differently; however, it may be more convenient to have the first element of the seed aligned with the first element of the read for the first vector. The next vectors are just the previous vectors padded by one 0-element from the left (as is done above). Or we may align the last elements of the seed and read and pad by 0-element from the right.

In any case, for a given seed, we form $(n_r-n_s+1)$ vectors of length $n_r$. We generate all possible combinations of $i_k$ indices. There are $n_m$ indices, and we want them to be different. Thus we get

3 $$\begin{aligned} C_{n_r}^{n_m} \equiv \frac{n_r!}{n_m! (n_r- n_m)!} \end{aligned}$$

such combinations. If, for any of these combinations, all $(n_r-n_m+1)$ vectors/rows contain at least one 1-element for chosen indices $i_k$, then the seed cannot be used. Otherwise, the seed meets the requirements, i.e. it is a valid seed.

Now we discuss how to perform seed validation as vector operations. As it is shown above, by padding seed *s* of length $n_s$ with 0-elements from the left and right, we create $(n_r - n_s+1)$ rows $L_p$ of length $n_r$. If there are $n_m$ mismatches, then we need to process $C_{n_r}^{n_m}$ cases. We create a vector *V* for each case, so all its elements are 1s except $n_m$ elements, which are 0-elements. For the first example, we choose elements 7 and 12, so one can write the corresponding $V_1$ vector as $V_1=\texttt{11111101111011111111}$. Then one needs to check that $V | L_p$ equals *V* for at least one index $p=1,\ldots ,(n_r-n_s+1)$. For the above example, we check $L_3$:

$$\begin{aligned} \begin{array}{lcl} V_1 &{}= &{} \texttt{11111101111011111111},\\ L_3 &{} = &{} \texttt{00110101011001011000},\\ V_1 | L_3 &{} = &{} \texttt{11111101111011111111}, \end{array} \end{aligned}$$

so we get $V_1 | L_3 = V_1$. Note that logical OR operations are bitwise, i.e., applied to each element of a vector.

The third example (elements 4 and 13) gives us vector $V_3 = \texttt{11101111111101111111}$, then for all vectors $L_p$, $p=1,\ldots ,6$, we find $V_3|L_p$:

$$\begin{aligned} \begin{array}{lclcl} V_3 | L_1 &{} = &{} \texttt{11111111111101111111} &{} \ne &{} V_3,\\ V_3 | L_2 &{} = &{} \texttt{11101111111111111111} &{} \ne &{} V_3,\\ V_3 | L_3 &{} = &{} \texttt{11111111111101111111} &{} \ne &{} V_3,\\ V_3 | L_4 &{} = &{} \texttt{11111111111101111111} &{} \ne &{} V_3,\\ V_3 | L_5 &{} = &{} \texttt{11111111111111111111} &{} \ne &{} V_3,\\ V_3 | L_6 &{} = &{} \texttt{11111111111111111111} &{} \ne &{} V_3. \end{array} \end{aligned}$$

Therefore, one cannot use the seed for the given combination of indices, and, as a result, it cannot provide us with full sensitivity. Only when the procedure is successful for all $C_{n_r}^{n_m}$ combinations, then the seed is valid.

It is clear that if we pad all vectors $L_p$ and *V* with 1-elements, then the validation criterion is still valid since $1 | 1 = 1$. Therefore we may use various SIMD intrinsics to perform logical OR operations on 128-bit numbers.

**Fig. 1:**
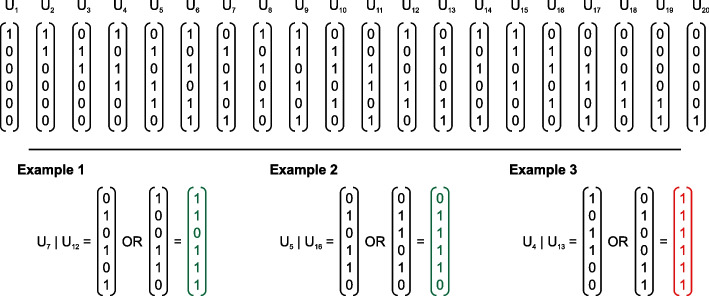
Seed validation based on columns. When the requirement is met, the row is in green colour, otherwise, in red colour

There is an alternative approach to validating seeds. We create $n_r$ binary vectors (columns) $U_t$, $t=1,\ldots , n_r$, of length $(n_r-n_m+1)$, see Fig. 1. As before, we may pad them with 1-elements to form numbers of a given length, e.g. 32-, 64- or 128-bit numbers. The task is to consider all $C_{n_r}^{n_m}$ combinations of columns $U_t$, perform logical OR operations, i.e.

4 $$\begin{aligned} U_{j_1} | U_{j_2} |\ldots | U_{j_{n_m}}, \end{aligned}$$

and check if the resultant column has all 1-elements (let us call it the *saturated* vector).

**Fig. 2:**
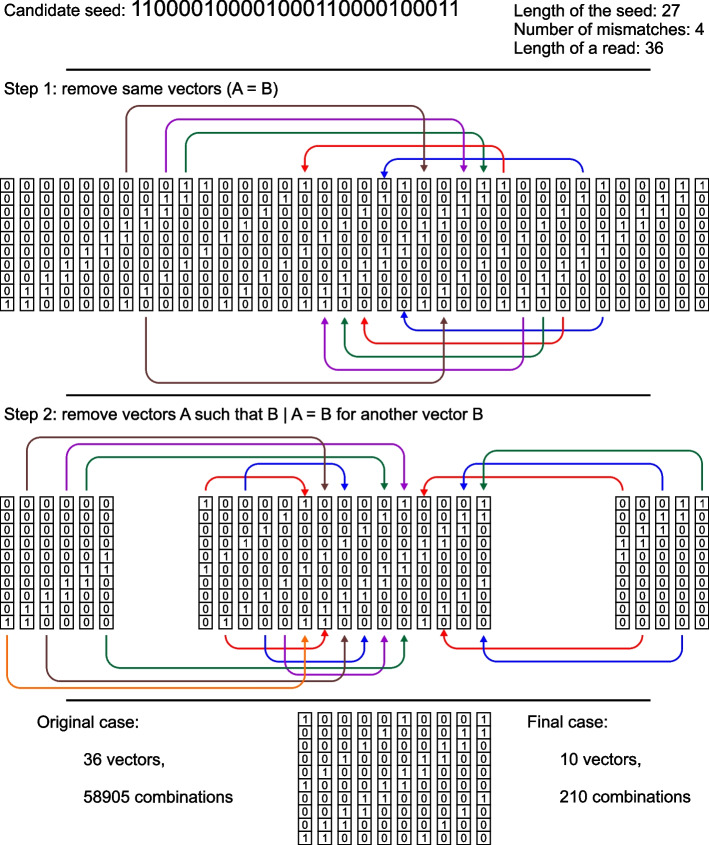
Reducing the number of combinations when checking seed’s validity

Now we try to reduce the number of columns. We may identify the same columns and leave only different ones (remove 10 out of 36 columns in the example in Fig. 2). So, as $n_m = 4$ and $n_r = 36$, then instead of $C^4_{36} = 58905$ combinations, we should check only $C^4_{26}=14950$. The next step is identifying those columns that are subsets of other columns. We denote $U_k \subset U_m$ if $U_k | U_m$ is $U_m$. This means that all 1-elements of column $U_k$ are also 1-elements of $U_m$ (however, there may be positions such that element *q* of $U_m$ is 1-element but element *q* of $U_k$ is 0-element). For example, $U_{14}=(\texttt{0010000100})^T$, $U_{19}=(\texttt{0010001100})^T$ and $U_{14}\subset U_{19}$. If there is a combination of vectors $U_k$ that includes $U_{14}$ and provides us with the saturated resultant vector, then the same combination but with $U_{19}$ also provides us with the saturated vector. So, we may exclude $U_{14}$. Removing columns that are subsets of other columns allows us to decrease the number of combinations further. For the example in Fig. 2, we excluded extra 16 columns, so the total number of combinations is $C^{4}_{36-10-16} = C^{4}_{10} = 210$, i.e. the number of combinations we need to check is 280 times fewer compared to the original case.

We may use similar approaches when forming resultant columns. Suppose $n_m > 2$. As $U_{i_1} | U_{i_2} = U_{i_2} | U_{i_1}$ and $U_{i_1} | U_{i_1} = U_{i_1}$, then we vary $i_1$ from 1 to $(n_r-1)$ and then $i_2$ from $(i_1 + 1)$ to $n_r$, so we consider $n(n-1)/2$ combinations. $U_t$ are binary vectors, so the resultant vectors or OR operations. So all vectors can be considered as numbers in binary representation and sorted in ascending/descending order. Likely, some of the $n(n-1)/2$ resultant vectors are the same, so we exclude them. Therefore by keeping resultant vectors for intermediate steps and checking if new vectors (i.e. $U_{i_3}$) are subsets of vectors from the set of intermediate resultant vectors (i.e. $U_{i_3} \subset \left( U_{i_1} | U_{i_2}\right)$), we may speed up processing.

### Seed expansion

The first and last elements of a seed are 1-elements. There is only one seed of length 1 (1), one seed of length 2 (11), two seeds of length 3 (101, 111), four seeds of length 4 (1001, 1011, 1101, 1111). Therefore for a read of length $n_r$, there are

5 $$\begin{aligned} 1 + 1 + 2 + 4 + \ldots + 2^{n_r-2} = 2^{n_r-1} \end{aligned}$$

seeds in total. As validation of each seed is also quite time-consuming, we should identify ways to reduce the number of candidate seeds. For example, seed 10001110100100011101 is valid for $n_r=30$ and $n_m=3$. Any subset of this seed is also valid (seeds of a lower weight). So, 1110100100011101 (the first four elements of the original seed are removed), 100011101001000111 (the last two elements), 10001010100100010101 (two random 1-elements are removed) are also valid seeds. Therefore, we can implement the following procedure.

1. Suppose we have already found all valid seeds of length less or equal to *k*.
2. We pad all these seeds with 0-elements from their right ends, so we get vectors of length *k*.
3. We concatenate them with 1-element (also at the right ends of the padded seeds).
4. Before applying the validation procedure, we form a seed by removing the first 1-element and all 0-elements from the left end and checking that the seed is in the list of valid seeds.

For example, if an original valid seed was 100111011 (of length 9), and we aim to find valid seeds of length 13, then the extended seed is 1001110110001, and we need to check if 1110110001 is already in the list of valid seeds.

Clear that a seed is valid if and only if the reverse seed (the order of elements is reversed) is valid. So, seeds 1001110110001 and 1000110111001 are valid (or are not valid) simultaneously.

### Periodic seeds

The above procedure allows us to reduce the number of candidate seeds. However, there are still a lot of seeds to be validated, so finding all possible seeds for a read’s length of more than 45 is very slow. However, we can observe a very important property. When for given $n_r$ and $n_m$ we find all seeds of maximum weight, then there are almost always *periodic* seeds such that

6 $$\begin{aligned} n_r = n_s + T - 1, \end{aligned}$$

where *T* is the size of the periodic block. Such seeds have a whole number $n_b$ of these periodic blocks and the “remainder” (the first $n_d > 0$ elements of the block), so $n_s = n_b \cdot T + n_d$.

For example, for $n_r=17$ and $n_m=3$ we get three pairs of seed: 111011, 1101000011, 1100100011 (and the reversed seeds). For the first seed, we have $\texttt{111011} = \texttt{1110} + \texttt{11}$, so $T=4$ and $n_s = 6$. We check that $n_s+T-1 = 6+4-1 = 9\ne 17 = n_r$. However, for the second seed we get $\texttt{1101000011} = \texttt{11010000} + \texttt{11}$, $T = 8$, $n_s=10$, so $n_s+T-1 = 10 + 8 -1 = 17 = n_r$. Thus, for four seeds Eq. (6) is met but for other two seeds it is not.

**Fig. 3:**
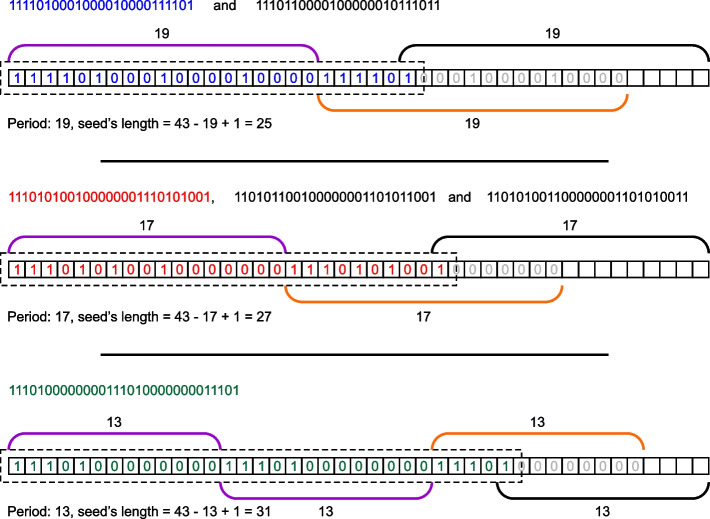
All seeds of maximum weight (12) found for reads of length 43, number of mismatches is 4. Reversed seeds are not shown

There may be seeds of different period *T* for the same length $n_r$ of a read. For example, in Fig. 3 there are seeds found for $n_r=43$, $n_m=4$. The maximum weight is $w=12$. We get two pairs of seeds for $T=19$, three pairs for $T=17$ and one for $T=13$.

Note that when one can find such seeds, other (shorter) seeds are often available. There are also several exceptions from the observation. For example, for some values of $n_r$ and $n_m$, we may have seeds such that (6) is valid for smaller values of $n_r$, e.g. $n_r-1$, $n_r-2$. However, there may be cases when the formula is not true for the best seeds. See Table 1. For example, if $n_r=16$, $n_m=5$ the best seeds are 1101 and 1011 ($n_s=4$ and $T=3$), so $n_s+T-1 = 4+3-1 = 6\ne 16$. However, for 80% of read lengths, formula (6) is valid for a given value of $n_r$, for 87% is true for smaller values of $n_r$.

**Table 1:** Exceptions from the observed formula

$n_m$	$n_r$, min	$n_r$, max	Exceptions ($n_r$ values)
2	12	42	no
3	11	42	11 ($\times$), 13 (12), 39 (38)
4	20	52	no
5	13	50	13 (12), 16 ($\times$), 17 ($\times$), 19 (18), 20 ($\times$), 47 (46)
6	14	53	15 (14), 17 (16), 19 (18), 23–26 ($\times$), 34 ($\times$), 38 ($\times$), 39 ($\times$), 47 ($\times$), 48 ($\times$), 52 ($\times$)
7	16	46	17 (16), 19 (18), 21–23 ($\times$), 25 (24), 26 ($\times$), 27 ($\times$), 29 (28), 43 ($\times$), 45 (44)
8	18	46	19 (18), 21 (20), 23–26 ($\times$), 28–32 ($\times$), 34 (33), 42–44 ($\times$)

If the formula is valid for shorter reads, then the corresponding $n_r$ is in parentheses, otherwise ($\times$)

**Fig. 4:**
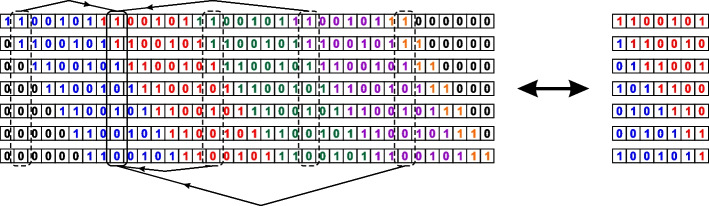
A seed is valid only when its periodic block is valid

Suppose there is a periodic seed such that $n_s = n_b T + n_d$ and formula (6) is true. We want to check if a seed is valid for a given $n_m$; see Fig. 4. We need to generate indices $j_k$, $k=1,\ldots ,n_m$ such that $1\le j_k\le n_r$ and check if the resultant column $U_{j_1} | U_{j_2} |\ldots |U_{n_m}$ is the saturated vector. A periodic block should meet the same requirements if the seed is valid. For this purpose, we choose $j_k$ such that $(1+ T)\le j_k \le 2T$. Now we assume that the resultant column for the periodic block is not the saturated one for all possible combinations of indices. For any index $j_k$, $1\le j_k\le n_r$, we may write down $j_k = j_k^* + m\cdot T$, where $(1+T)\le j_k^*\le 2T$ and *m* is an integer number. Therefore $U_{j_k} \subset U_{j^*_k}$ and instead of $U_{j_k}$ we may consider $U_{j_k^*}$.

### Periodic blocks

We validate a periodic block as the whole seed:

- generating all possible combinations of indices $j_k$, $k=1,\ldots ,n_m$, such that $1\le j_k\le T$;
- checking if the resultant column is the saturated one.

**Fig. 5:**
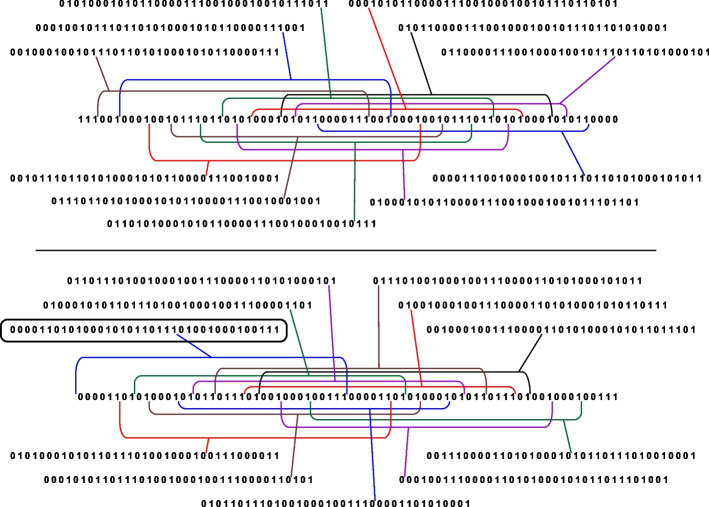
Equivalent periodic blocks generated for block 1011101001000100111000011010100010101. Only blocks with the first 0-element and last 1-element are shown. The bottom part is for the reverse block. The circled block is the one we choose from the group

A periodic block is valid if and only if its reverse is valid. We may also perform a cyclic rotation of elements of the block; those new blocks are valid at the same time. As a result, we have groups of 2*T* periodic blocks of length *T* (some may be identical) that are valid/not valid simultaneously. We call them *equivalent* blocks. Therefore it is enough to consider the validity of only one block. To reduce the number of blocks to be considered, we require that the first element is always 0-element and the last element is always 1-element. See examples of equivalent blocks generated for a given periodic block in Fig. 5.

We need a procedure to choose only one block instead of 2*T* blocks. Of course, for some blocks (e.g. 00101101), there may be cases of identical blocks obtained via reversion/cyclic rotation. By varying indices $j_k$, we create patterns of 0-elements. Those patterns should be within the periodic block (or one of the equivalent blocks obtained by cyclic rotation) since there should be a row such that the corresponding elements of the chosen columns are all 0-elements. Therefore each periodic block must have a contiguous chunk of $n_m$ 0-elements. Thus when we generate various periodic blocks, we may always assume that the first $n_m$ elements are 0-elements.

To choose one block, we pick up the one with the highest number of 0-elements at the beginning. Among blocks obtained for the reverse block, there will also be a block with the maximum number of 0-elements. Then, we need to perform a further comparison. We may count the number of 1-elements in contiguous blocks from the left/right side of the zero-block and choose the block with fewer 1-elements. If the contiguous blocks are the same, then consider neighbouring blocks of 0-elements (and choose the smallest one). We repeat the procedure if it is needed.

Algorithm 1 allows us to validate a block, i.e. check if it can be used for reads of at most $n_m$ mismatches. Suppose we have a binary vector *s* of length *T*. By performing a cyclic rotation of the vector when the last *n* elements of the vector become the first elements of a new vector (the order of elements is preserved), we generate *T* vectors $\eta _i$ of length *T*. Then we just need to consider all possible combinations of $n_m$ vectors and check if the resultant vector obtained by applying logical bitwise OR operation is the saturate vector, i.e. the vector with all 1-elements. For this purpose we create $n_m$ indices $k_p$, $p=1,\ldots , n_m$. When we have $n_m$ binary vectors and apply OR operation, then the order of vectors does not matter. Therefore we may assume that $k_p<k_{p+1}$, $p=1,\ldots ,(n_m-1)$. So, we initialise the indices as $k_p=p$, $p=1,\ldots ,n_m$, and to create a new set of indices we increment the last index $k_{n_m}$. When $k_{n_m}$ reaches the maximum value (*T*), we start incrementing the previous index $k_{n_m-1}$ and reset $k_{n_m}$ to the smallest value permitted. This procedure is applied in a similar way to other indices. Clearly, that if after incrementing of $k_p$ by one we get $k_{p}$ equal to $(T+p-n_m+1)$, then we should increment $k_{p-1}$ are reset all other indices $k_p, k_{p+1}, k_{p+2}, \ldots$.
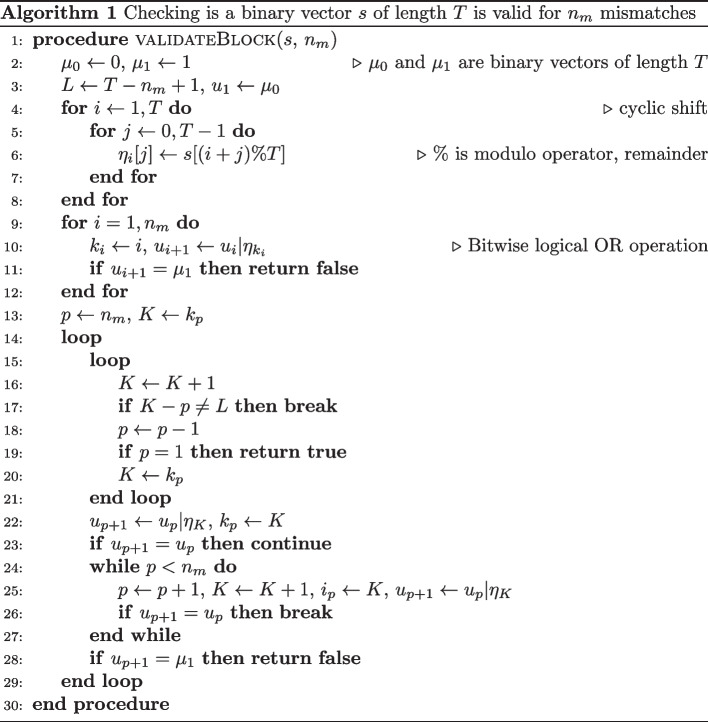


The algorithm should stop when $p-1=0$. However, we may stop earlier when $p-1=1$. This means that we may actually always set $k_1=1$. Suppose that we have an arbitrary combination of ordered indices $k_p$, $p=1,\ldots , n_m$ and form a resultant vector *v* after applying bitwise OR operations. However we may create another set of indices ${\bar{k}}_p$ such that ${\bar{k}}_p\equiv (k_p-k_1)\%T + 1$. The resultant vector found for $k_p$ indices is the resultant vector found for ${\bar{k}}_p$ indices but after the cyclic shift by $(k_1-1)$ elements was performed. So, both resultant vectors are (or are not) saturated at the same time.

We store intermediate $n_m$ resultant vectors $u_p$ and perform bitwise OR operation with the corresponding $\eta _{k_p}$ to find $u_{p+1}$. If $\eta _{k_p}$ is a subset vector for $u_p$, then as it was discussed in previous subsections we may ignore $\eta _{k_p}$ and consider other vectors.

As we want to find seeds of maximum weight, it is reasonable to find periodic blocks of maximum weight. Suppose there are blocks found for weight *w*. By replacing 1-elements with 0-elements we form periodic blocks of weight $(w-1)$. However, there may also be blocks of weight $(w-1)$ that cannot be formed by replacing 1-elements in blocks of weight *w*. For example, there are five blocks for $T=11$, $n_m=2$ and weight $w = 7$: 00011011111, 00101011111, 00101110111, 00101111011, 00110101111. Block 00010110111 of weight $w=6$ cannot be formed from those blocks. Seeds are formed of an integer number of periodic blocks and a “remainder”. Therefore, there is a possibility that the best seeds are formed of blocks of non-maximum weight. However, when we generated seeds of non-maximum weight, they never formed seeds of weight larger than seeds formed from maximum-weight blocks.

Thus to generate all seeds of maximum weight for blocks of length *T* and $n_m$ mismatches we set maximum weight *w* (at most $T-n_m$) and consider all binary vectors such that the first $n_m$ elements are zeros and the last element is one. Then validate these seeds using Algorithm 1. If no seeds are found for a given *w*, then reduce *w* by 1 and generate a new set of candidate vectors.

All ideas mentioned above allow us to reduce the number of blocks to be validated. It is also possible to parallelise processing so each CPU thread validates only specific seeds from a pre-generated list. Together with SIMD instructions for validation steps, we sped up the generation of periodic blocks. It may take less than a second for $n_r < 35$, however, finding periodic blocks for $n_r\approx 50$ may still take hours as trillions of blocks should be validated.

Algorithm 1 for validation of seed blocks can be implemented on various computational architectures. The authors implemented it using various SIMD operations. The algorithm’s performance will depend on input parameters and possible optimisation done by a compiler and specific elementary operations. This algorithm has five major operations: OR, XOR, binary shift and extraction of a number applied to 128-bit numbers (all SIMD operations) and elementary addition (as increment/decrement operations) for 32-bit numbers. We may ignore the time spent on other operations. In principle, the number of XOR, binary shift and extraction operations is the same for this algorithm. So, validating a single block will take about $O(n_{\text{OR}} + 3 n_{\text{XOR}} + n_{add})$ elementary operations. Thus, we mention only OR ($n_{\text{OR}}$), XOR ($n_{\text{XOR}}$) and elementary addition ($n_{add}$) operations. These numbers are in Table 2. We may see that the ratio $n_{\text{XOR}}/n_{\text{OR}}$ is almost the same ($\approx 0.92$), while $n_{add}/n_{\text{OR}}$ is increased with $n_m$ (from 0.3 for $n_m=2$ to 0.9 for $n_m=9$). The total number of OR operations may differ for different *T*; however, we usually see a five-fold increase when $n_m$ is increased by one. The main issue when generating all possible seed blocks is exponential (as a function of *T*, different functions for different values of $n_m$, e.g. $\sim T^{12}$ or $T^{20}$) increase of the number of blocks to be validated even after various procedures to reduce the number of candidate blocks.

**Table 2:** Number of operations used for Algorithm 1: number of mismatches ($n_m$), length of a periodic block (*T*), total number of seeds to be validated (# tests), averaged numbers of SIMD OR ($n_{\text{OR}}$), XOR ($n_{\text{XOR}})$ and standard addition ($n_{add}$) operations for each binary block as an absolute number or as percentage of $n_{\text{OR}}$ operations

$n_m$	*T*	# tests	$n_{\text{OR}}$	$n_{\text{XOR}}$	% of $n_{\text{OR}}$	$n_{add}$	% of $n_{\text{OR}}$
2	30	2391	218	194	89	74	34
2	35	6259	174	150	86	51	29
2	40	66,542	206	182	88	67	33
2	50	330,586	161	137	85	45	28
2	60	7,780,954	169	145	86	49	29
3	25	6296	459	419	91	188	41
3	29	88,578	955	906	95	464	49
3	33	595,263	387	350	90	145	37
3	38	14,780,813	571	532	93	241	42
4	25	4989	2858	2724	95	1630	57
4	29	68,931	3405	3274	96	1893	56
4	33	966,747	4292	4153	97	2361	55
4	38	32,240,220	1501	1437	96	730	49
5	25	780	105,377	97,501	93	77,081	73
5	29	13,668	30,507	28,945	95	19,946	65
5	33	229,001	14,869	14,342	96	8865	60
5	38	9,574,775	6432	6238	97	3566	55
6	25	283	80,726	74,048	92	61,606	76
6	30	7520	32,220	30,638	95	21,071	65
6	35	200,437	29,377	28,254	96	18,128	62
6	38	502,937	320,811	305,715	95	208,510	65
7	25	83	176,631	158,692	90	146,921	83
7	30	6205	7026	6672	95	4362	62
7	33	5173	717,404	665,819	93	526,252	73
7	38	448,976	31,566	30,395	96	19,488	62
8	30	728	92,676	85,827	93	70,045	76
8	35	1909	32,199,261	29,015,989	90	26,230,401	81
9	30	177	2,632,939	2,267,998	86	2,547,863	97
9	35	1784	1,722,183	1,556,851	90	1,419,823	82

**Fig. 6:**
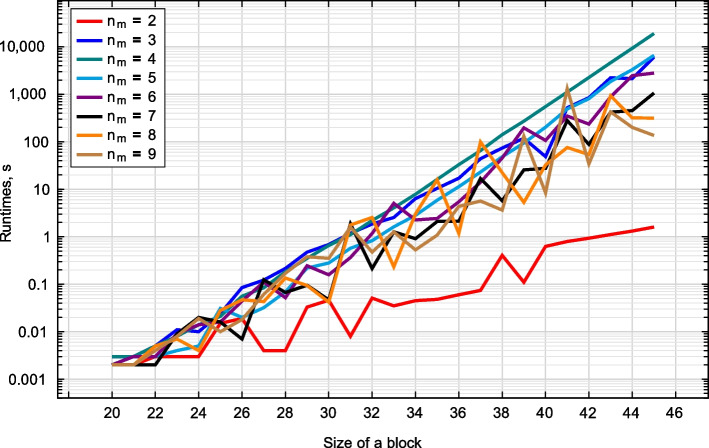
Times required to generate periodic blocks of maximum weight for a given number of mismatches $n_m$ and a known block size

The times needed to generate periodic blocks depend on CPU architecture. Run times for Intel Core i5-9600K processor (6 cores, 12 threads, base frequency 3.70 GHz) can be found in Table 3 and Fig. 6. One may see that calculations for $n_m=4$ are the slowest, and in this case, the run times increase exponentially as $2.85\cdot 10^{-10}\cdot 2.03^T$ (in seconds). If blocks are less than 30, we can perform all calculations in less than a second. When blocks have 40 elements, we need an hour to complete the task, while 50 elements may require us to spend a week or use a CPU with tens of cores.

**Table 3:** Calculation times (in seconds) for Algorithm 1 for Intel Core i5-9600K processor ($n_m$ is the number of mismatches, *T* is the size of the periodic block)

*T*	$n_m=2$	$n_m=3$	$n_m=4$	$n_m=5$	$n_m=6$	$n_m=7$	$n_m=8$	$n_m=9$
25	0.02	0.02	0.02	0.03	0.02	0.02	0.03	0.01
30	0.05	0.69	1.10	0.28	0.16	0.05	0.04	0.35
35	0.05	10.45	16.11	5.87	2.43	2.09	15.82	1.08
38	0.41	73.85	141.16	48.25	45.46	5.73	22.41	3.64
40	0.63	48.71	551.05	203.14	106.70	27.52	32.51	8.40
42	0.93	849.18	2257.21	809.38	235.92	88.09	53.71	34.19
45	1.60	6105.13	19,009.34	6611.98	2797.29	1058.58	315.64	136.01

### Forming periodic spaced seeds

The final step of PerFSeeB approach (periodic full sensitivity blocks) is to form spaced seeds of maximum weight. For this purpose, we consider all periodic blocks formed for a given number $n_m$ of mismatches. As $n_s = n_b T + n_d$, $n_b \ge 1$, $1 \le n_d < T$, and $n_r = n_s + T-1$, then $n_r = n_b T + n_d + T - 1 = (n_b + 1)T +(n_d-1) \ge 2T$ or $T \le n_r/2$. For each periodic block we form all equivalent blocks and check if

1. the first symbol of the equivalent block is 1-element,
2. the $n_d$-th element is also 1-element.

In principle, there is no need to consider blocks obtained from the reverse periodic block as the final seed will be a reverse of seeds formed from the original block. Once the seed is formed we count its weight. By processing all periodic blocks we find seeds of maximum weight. The detailed procedure is shown in Algorithm 2. We assume that we already have files with periodic blocks found for a set of lengths, from $T_{min}$ to $T_{max}$, and valid for at most $n_m$ mismatches. After application of Algorithm 2 we get a list $\Omega$ of $n_{sol}$ periodic seeds of maximum weight. Note that for the same weight *w* and the number of mismatches $n_m$ we may have seeds made of periodic blocks of different length/weight. Time required to run Algorithm 2 is the sum of times to process all blocks available for given values of $n_m$ and *T*. While the number of valid blocks is different for a pair of $n_m$ and *T* (from single entries to a million), it takes at most 5 s on Intel’s i5-9600K CPU to find the best seed for a given length of a read.
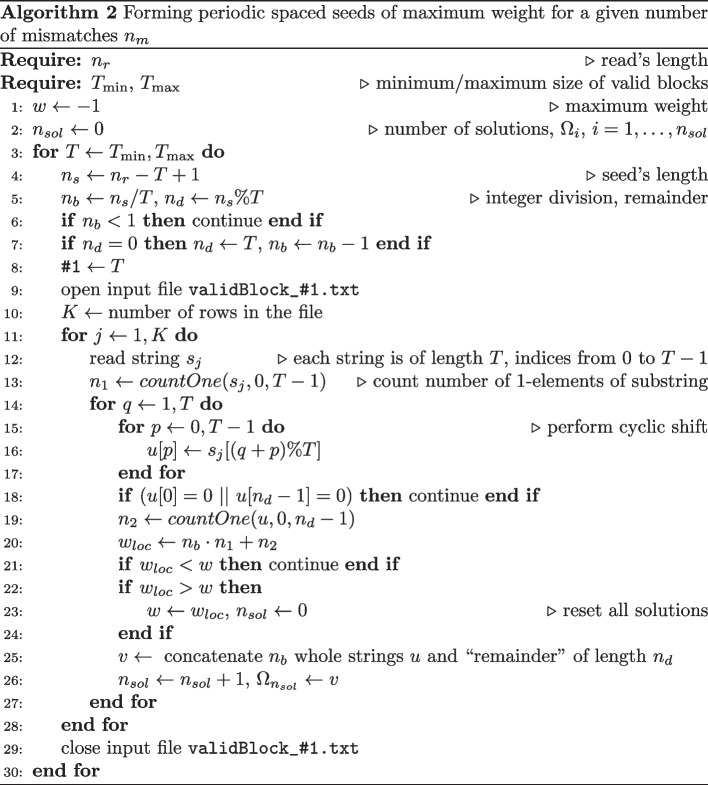


## Results

### Periodic seeds and their properties

We have written a code to generate the best periodic blocks of size *T* for a given number $n_m$ of mismatches. A user can specify an initial number $n_1$ of 1-elements in those blocks. If no valid blocks are found for a given $n_1$, then the value of $n_1$ is incremented by one and the procedure restarts until at least one valid block is found. The code was applied for $n_m$ values from 2 to 9, and for *T* values from 10 to 50 (and from 10 to 70 for $n_m=2$). Maximum weight $n_1$ and density of best blocks (defined as $n_1/T$) are shown in Table 4.

**Table 4:** Maximum weight $n_1$ and density $\rho$ of periodic blocks (period *T*, number of mismatches $n_m$)

*T*	$n_m = 2$		$n_m = 3$		$n_m = 4$		$n_m = 5$		$n_m = 6$		$n_m = 7$		$n_m = 8$	
	$n_1$	$\rho , \%$	$n_1$	$\rho , \%$	$n_1$	$\rho , \%$	$n_1$	$\rho , \%$	$n_1$	$\rho , \%$	$n_1$	$\rho , \%$	$n_1$	$\rho , \%$
10	6	60.0	4	40.0	3	30.0	2	20.0	1	10.0	1	10.0	1	10.0
11	7	63.6	5	45.5	3	27.3	2	18.2	1	09.1	1	9.1	1	9.1
12	8	66.7	5	41.7	3	25.0	3	25.0	2	16.7	2	16.7	1	8.3
13	9	69.2	6	46.2	4	30.8	3	23.1	2	15.4	1	7.7	1	7.7
14	9	64.3	6	42.9	4	28.6	4	28.6	3	21.4	2	14.3	1	7.1
15	10	66.7	8	53.3	5	33.3	4	26.7	2	13.3	2	13.3	2	13.3
16	11	68.8	8	50.0	5	31.3	4	25.0	3	18.8	3	18.8	1	6.3
17	12	70.6	8	47.1	6	35.3	4	23.5	3	17.6	2	11.8	2	11.8
18	13	72.2	9	50.0	6	33.3	5	27.8	3	16.7	3	16.7	3	16.7
19	14	73.7	10	52.6	7	36.8	5	26.3	4	21.1	3	15.8	2	10.5
20	14	70.0	10	50.0	8	40.0	6	30.0	4	20.0	4	20.0	3	15.0
21	16	76.2	11	52.4	8	38.1	6	28.6	5	23.8	4	19.0	4	19.0
22	16	72.7	12	54.5	8	36.4	7	31.8	5	22.7	5	22.7	3	13.6
23	17	73.9	12	52.2	9	39.1	7	30.4	5	21.7	4	17.4	3	13.0
24	18	75.0	14	58.3	9	37.5	8	33.3	5	20.8	5	20.8	4	16.7
25	19	76.0	14	56.0	10	40.0	7	28.0	6	24.0	5	20.0	4	16.0
26	20	76.9	14	53.8	10	38.5	9	34.6	6	23.1	6	23.1	4	15.4
27	21	77.8	15	55.6	11	40.7	9	33.3	6	22.2	5	18.5	5	18.5
28	22	78.6	16	57.1	11	39.3	9	32.1	8	28.6	6	21.4	4	14.3
29	22	75.9	16	55.2	12	41.4	9	31.0	7	24.1	6	20.7	5	17.2
30	23	76.7	17	56.7	13	43.3	10	33.3	8	26.7	8	26.7	6	20.0
31	25	80.6	18	58.1	16	51.6	10	32.3	8	25.8	6	19.4	5	16.1
32	25	78.1	19	59.4	14	43.8	11	34.4	8	25.0	8	25.0	5	15.6
33	26	78.8	20	60.6	14	42.4	11	33.3	8	24.2	7	21.2	7	21.2
34	27	79.4	20	58.8	15	44.1	12	35.3	9	26.5	8	23.5	6	17.6
35	28	80.0	21	60.0	15	42.9	12	34.3	10	28.6	8	22.9	6	17.1
36	29	80.6	22	61.1	16	44.4	13	36.1	10	27.8	9	25.0	8	22.2
37	30	81.1	22	59.5	17	45.9	13	35.1	10	27.0	8	21.6	6	16.2
38	30	78.9	23	60.5	18	47.4	14	36.8	10	26.3	10	26.3	7	18.4
39	32	82.1	24	61.5	18	46.2	14	35.9	10	25.6	9	23.1	9	23.1
40	32	80.0	27	67.5	18	45.0	14	35.0	11	27.5	10	25.0	8	20.0
41	33	80.5	25	61.0	19	46.3	14	34.1	11	26.8	9	22.0	8	19.5
42	34	81.0	26	61.9	19	45.2	16	38.1	13	31.0	11	26.2	10	23.8
43	35	81.4	26	60.5	20	46.5	15	34.9	12	27.9	10	23.3	8	18.6
44	36	81.8	28	63.6	21	47.7	16	36.4	12	27.3	11	25.0	9	20.5
45	37	82.2	28	62.2	21	46.7	17	37.8	13	28.9	11	24.4	10	22.2
46	38	82.6	29	63.0	22	47.8	17	37.0	13	28.3	12	26.1	9	19.6
47	39	83.0	30	63.8	23	48.9	17	36.2	13	27.7	11	23.4	9	19.1
48	40	83.3	31	64.6	23	47.9	18	37.5	14	29.2	14	29.2	10	20.8
49	41	83.7	31	63.3	26	53.1	18	36.7	15	30.6	12	24.5	10	20.4
50	42	84.0	32	64.0	24	48.0	19	38.0	15	30.0	14	28.0	10	20.0

**Fig. 7:**
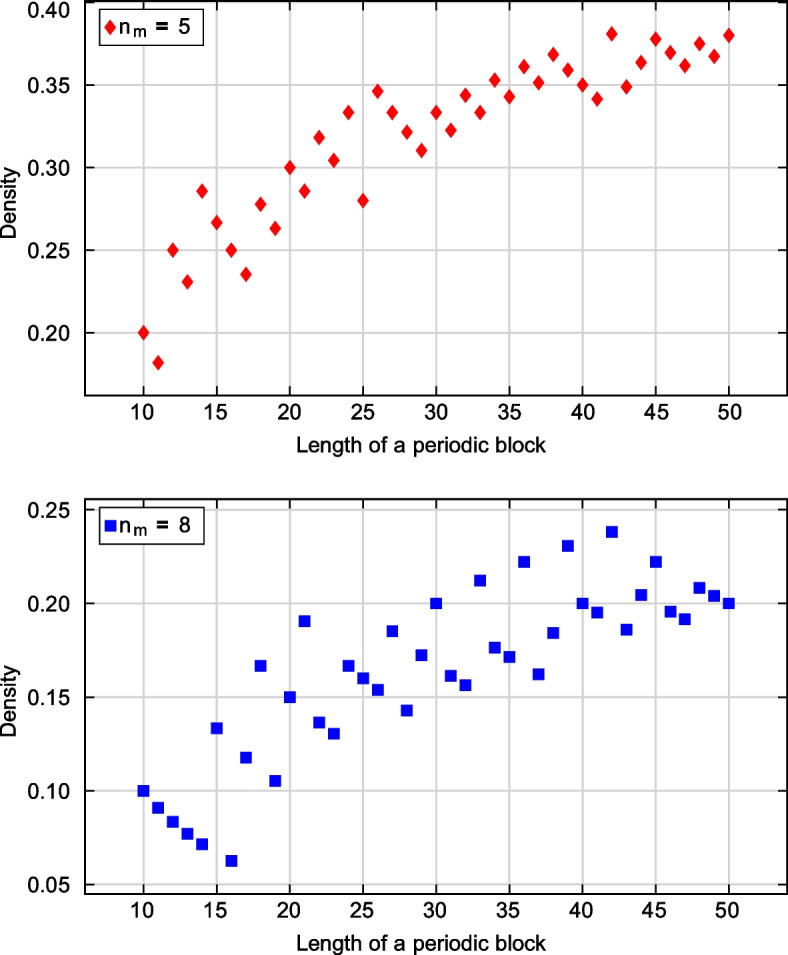
Maximum numbers of 1-elements per length of periodic blocks, $n_m=5$ and $n_m=8$

Note that while values $n_1$ and $\rho$ tend to increase with the block size, they are not monotonic in general. See examples for $n_m=5$ and $n_m=8$ in Fig. 7 (more figures are in Additional file 1).

When all maximum-weight blocks are found for a given range of *T*, we may find periodic spaced seeds of the maximum weight for any given value of $n_r$. Of course, there may be cases of seeds composed of periodic blocks of different sizes. For example, if the length of reads is $n_r = 35$, the maximum weight of possible seeds is $w=17$ and there are seeds formed for blocks of sizes 7, 10, 11, 13, e.g.

- $$T=7$$, $$n_b=4$$, $$n_d=1$$, $$b=\texttt{1011100}$$, $$s=\texttt{10111001011100101110010111001}$$;
- $$T=10$$, $$n_b=2$$, $$n_d=6$$, $$b=\texttt{1101111000}$$, $$s=\texttt{11011110001101111000110111}$$;
- $$T=11$$, $$n_b=2$$, $$n_d=3$$, $$b=\texttt{11111000110}$$, $$s=\texttt{1111100011011111000110111}$$;
- $$T=13$$, $$n_b=1$$, $$n_d=10$$, $$b=\texttt{1011111011100}$$, $$s=\texttt{10111110111001011111011}$$.

**Fig. 8:**
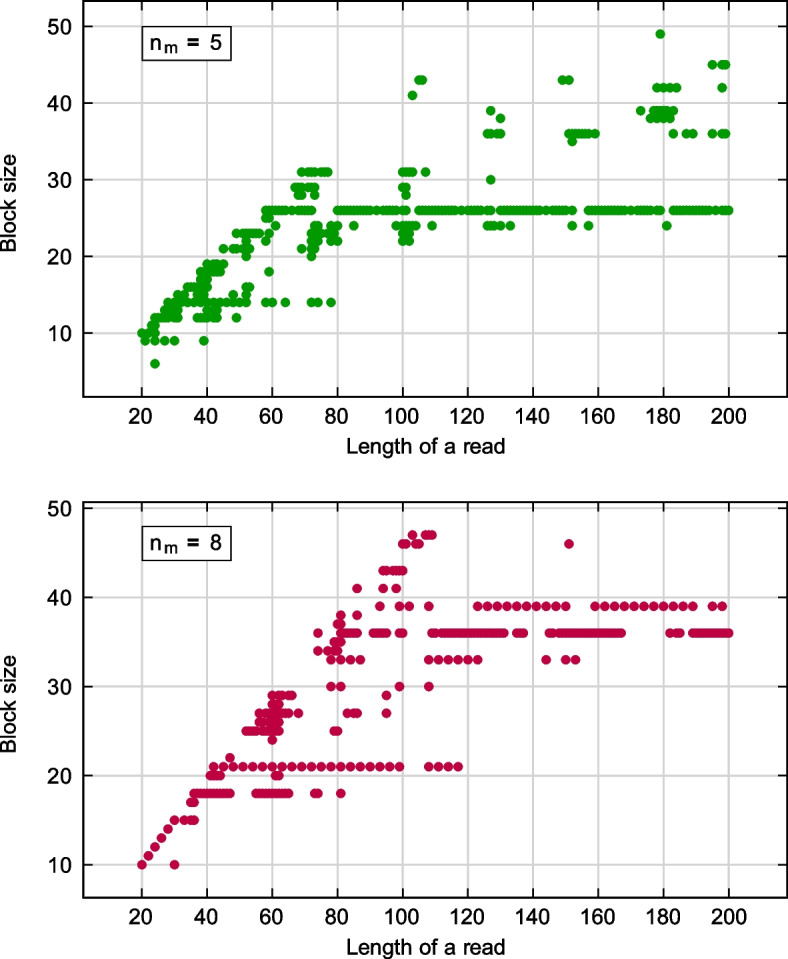
Sizes of best periodic blocks for a given length of reads

For a given length $n_r$ of reads, we plot sizes of corresponding periodic blocks, see Fig. 8. We see that sizes of best blocks tend to work in a specific range of $n_r$ values. Best blocks are often those having peak density values. For example, if $n_m=8$, the most frequent block sizes are $T=18, 21, 33, 36, 39$ (Fig. 8), we see peak density values for them in Fig. 7. Note that values of *T* also increase with the length of reads. So, while we can find seeds for any $n_r$, they may not be the densest as there we have not generated periodic blocks for large values of *T*.

The final goal is to set the weight *w* of seeds, then find the minimum length of reads that have valid seeds of this weight and choose the longest seeds when several seeds are available. In Table 5, we present examples of these seeds for weights *w* (multiples of 8). As seeds may be very long, we show only periodic blocks, number $n_b$ of these blocks and values of “remainders” $n_d$; see Tables 6 and 7. The complete list of spaced seeds found for various values of $n_r$ and *w* ($n_r \le 400$, $w \le 320$) is in Additional file 1. Users can generate their seeds using codes (https://github.com/vtman/PerFSeeB).

**Table 5:** Various longest periodic spaced seeds of the maximum weight for a given number of mismatches $n_m$ and weight *w*

$n_m$	*w*	$n_r$	Longest spaced seed
2	16	32	10111001011100101110010111
2	20	39	101110010111001011100101110010111
2	24	44	11111100110101111110011010111111
2	28	51	11011111000110111110001101111100011011111
2	32	56	11111011100101111101110010111110111001011111
2	36	62	10111110111001011111011100101111101110010111110111
2	40	68	11111011100101111101110010111110111001011111011100101111
2	44	73	1111101110010111110111001011111011100101111101110010111110111
3	16	41	100110101111000100110101111
3	20	48	1111000100110101111000100110101111
3	24	56	100110101111000100110101111000100110101111
3	28	63	1111000100110101111000100110101111000100110101111
3	32	71	100110101111000100110101111000100110101111000100110101111
3	36	78	1111000100110101111000100110101111000100110101111000100110101111
4	16	54	11110010000001000111100100000010001111
4	20	64	1101110100000010000110111010000001000011011101
4	24	70	1110111110010011000010110101000111011111
4	28	79	1100111110001101110101000010010110011111000110111
4	32	88	1001011001111100011011101010000100101100111110001101110101
4	36	96	111110001101110101000010010110011111000110111010100001001011001111
5	16	62	1110011010100000010010000011100110101
5	20	76	1100011010111110000000000000000110001101011111
5	24	86	1110011010100000010010000011100110101000000100100000111001101
6	16	75	10011010111100000000000000000000100110101111
6	20	91	1000011100000010000011000010100001110000001000001100001010000111
6	24	102	110011010101111110000000000000000000000000011001101010111111
7	16	82	10000010100010001010101000000010000010100010001010101
7	20	96	1010101000000010000010100010001010101000000010000010100010001010101

**Table 6:** Blocks for spaced seeds ($n_m = 2$)

*w*	$n_r$	Periodic block	$n_b$	$n_d$
16	32	1011100	3	5
24	44	1111110011010	2	6
32	56	1111101110010	3	5
40	68	1111101110010	4	4
48	80	1111101110010	5	3
56	91	1110110111111111000	3	16
64	102	1111111110001110110	4	8
72	112	111111111010111100110	4	8
80	123	110111111111010111100	4	19
88	133	111111111010111100110	5	8
96	144	110111111111010111100	5	19

**Table 7:** Blocks for spaced seeds ($n_m = 6$)

*w*	$n_r$	Periodic block	$n_b$	$n_d$
16	75	10011010111100000000000000000000	1	12
24	102	1100110101011111100000000000000000000000000	1	17
32	133	1000001100001010000111000000	3	22
40	161	1000001100001010000111000000	4	22
48	188	1100110101011111100000000000000000000000000	3	17
56	213	111100000010000011000010100000110001001000	4	4
64	239	110000011000010100001110000111000000100000	4	30
72	266	100001110000111000000100000110000011000010	5	15
80	295	110000011000010100001110000111000000100000	6	2
88	316	110000101000011100001110000001000001100000	6	23
96	343	100011110000001000001100001010000011000100	7	8

**Fig. 9:**
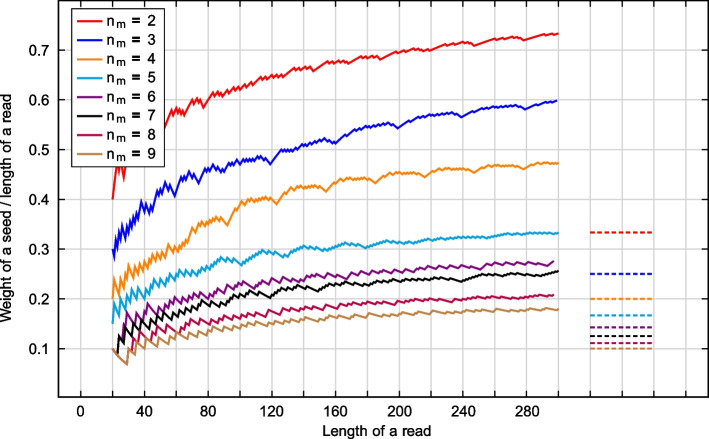
Weights of the best spaced seeds per reads’ lengths (solid lines). Maximum ratios for contiguous seeds (dashed lines)

We plot weights of the best seeds as a function of reads’ length in Fig. 9. Based on lemmas in [32], it is possible to show that for a contiguous seed of weight/length *q*, the minimum length of reads when the seeds are valid is $K=q(n_m +1)$, so the ratio is $r = q/K\rightarrow r_{\infty }=\frac{1}{n_m+1}$ when $q\rightarrow \infty$. The corresponding values of $r_{\infty }$ are also shown in Fig. 9 (dashed lines). Depending on values of $n_m$ and $n_r$, the spaced seeds are denser (per read’s length) compared to contiguous seeds by 20–68% ($n_r = 50$), 40–95% ($n_r = 100$), 60–113% ($n_r = 150$), 70–128% ($n_r = 200$).

### Other seeds

To compare the quality of spaced seeds generated with the PerFSeeB approach, we present a list of the most popular seeds in Tables 8 and 9. These seeds are generated for given sensitivity levels. When there were several seeds presented in a paper, we chose a seed obtained for the highest sensitivity levels. We may see that the seeds are usually relatively short and of smaller weight. Unlike the PerFSeeB approach, the other algorithms did not usually put any restrictions on the number of mismatches. Several seeds were generated for a multiple-seed approach.

**Table 8:** Examples of most popular spaced seeds

[33]	Diamond	[34]	BFAST
$D_1$	111101011101111	$B_1$	1111111111111111111111
$D_2$	111011001100101111	$B_2$	1111010010110110101110010110111011
$D_3$	1111001001010001001111	$B_3$	11111011011101111011111111
$D_4$	111100101000010010010111	$B_4$	1011110101101001011000011010001111111
		$B_5$	1111101110111010100101011011111
[8]	PatternHunter	$B_6$	10111001101001100100111101010001011111
$P_1$	111010010100110111	$B_7$	111111100101001000101111101110111
[17]	PatternHunter II	$B_8$	111101101011011001100000101101001011101
$P_2$	111100110010100001011	$B_9$	11110101110010100010101101010111111
$P_3$	110100001100010101111	$B_{10}$	1111011010001000110101100101100110100111
$P_4$	1110111010001111		
		[35]	rasbhari
[36]	Quadratic Residues	$R_1$	1111011110011010111110101011011
$Q_1$	101100001	$R_2$	1110101011101100110100111111111
$Q_2$	1011000010101111001	$R_3$	1111110101101011100111011001111
[37]			
$S_1$	111111111100011101100100100111010011100010100101000010100110000101111000000011		
$S_2$	11110111111011001111000110101100111010110000001110100011010010100111110011		
$S_3$	111011010100110101100100100101010110001		
$S_4$	11111111111111110001111111000011001100011100111100001110000000111101110000011		
$S_5$	1100000101011110100100000000110111111111100011000010111110010111011101011000101		
$S_6$	1110001000011110110101100000100010000111010110110110111101011011000100100101011

**Table 9:** Spaces seeds for MegaBLAST [38]

$M_1$	1111101101011001101100011110010101011101111
$M_2^1$	111111010111011110111011011011101111
$M_2^2$	11111101110011001101001011010111010111111
$M_4^1$	11111110111110111101111101111111
$M_4^2$	11111101101011011100100011111001010111111
$M_4^3$	11110111010011001010111101101110011110111
$M_4^4$	11111010111000111011011010001011111101111
$M_8^1$	11111010111011101111011110111011111
$M_8^2$	11100111111010111101100101111101110111
$M_8^3$	111101111011111000110010110101111101111
$M_8^4$	1111111001011110110011101001110110101111
$M_8^5$	11110110111010110110101011100011011011111
$M_8^6$	11111011011100101000111111101100010111111
$M_8^7$	11110101100110011111010111001001111011111
$M_8^8$	11101110100100110111110001111110101101111
$M_{16}^1$	11111101111011111011111011111111
$M_{16}^2$	11101011111101101111111110011110111
$M_{16}^3$	111110111110111110001101110111011111
$M_{16}^4$	111101110111111011110100011110111111
$M_{16}^5$	111111100011011110111011110111110111
$M_{16}^6$	1111110111110101011011011011100111111
$M_{16}^7$	11110111011001001111011111110101011111
$M_{16}^8$	111111011011110010011110101010011111111
$M_{16}^9$	111110111100101110010111101101101101111
$M_{16}^{10}$	1111100111011101111101001110010010111111
$M_{16}^{11}$	1111011011011111110100011010110101101111
$M_{16}^{12}$	11110101101110110000111001110110111101111
$M_{16}^{13}$	11111110010010111111011101001001110101111
$M_{16}^{14}$	11110111010100011101101000111111100111111
$M_{16}^{15}$	11111011000110100110001011111111011011111
$M_{16}^{16}$	11111011110101100011101110000111010111111

### Comparison of seeds

To estimate performance characteristics, we develop software tools for real reference sequences and reads. For a reference sequence, we choose the T2T data [30]. Then, we randomly selected a relatively small dataset (ERR263486 [39], part of the 1000 Genomes Projects [40]): it is a paired-end data, and we use only the first dataset, ERR263486_1.

1. There are 1734496 reads of length 100; we removed reads containing N symbols. Thus we get 1731924 reads. For each read, we also create its counterpart, i.e., flip the sequence and change $\texttt{A} \leftrightarrow \texttt{T}$, $\texttt{C} \leftrightarrow \texttt{G}$, so for ATCCCAAAGTGCTTT we generate AAAGCACTTTGGGAT. Therefore we use 3463848 sequences.
2. For a given seed, we generate the corresponding library of records (“key”, “value”) where “key” is the position within the reference sequence and sort it by “values”.
3. For a chosen seed of length $n_s$ we create $(101- n_s)$ “values” for each read. For each “value” we find the list of all “keys” in the library (if the “value” is present). Knowing the relative position of the seed to the read, we use the found seed to create a new list. All elements of this list are possible candidate positions at the start of the read. As we have at most $(101- n_s)$ such lists, we merge them into one list and remove all duplicated entries. We count the total number of possible candidate positions of the read with respect to the reference sequence. Those candidate positions may be used for proper read alignment. Ideally, we do not want to miss any position that may provide us with proper alignment (accounting for known restrictions), but at the same time, we what to keep the number of these positions to a minimum to increase the performance of a sequence alignment code.
4. Our goal is to see how seeds perform under our restrictions (when only at most a given number of SNPs is possible). We check all candidate positions for each read and its counterpart and find the maximum number of matches. If two seeds are to be compared, then the best seed should provide us with more matches. Of course, there may be several positions when the number of matches attains its maximum value (possible for the given seed). If two seeds provide the same maximum number of matches for a read, then the best seed should provide us with a longer list of the best candidate positions.

To assist a reader, we developed software tools to deal with users’ seeds and provide them with lists of all and best candidate positions and information about the total number of items in the lists and the maximum number of matches attained. The software is also available at (https://github.com/vtman/PerFSeeB):

1. **fna2bin** and **ref2m128**—convert input FNA file for the T2T reference data into binary files;
2. **fastq2bin** and **bin2m128**—convert FASTQ files into binary files;
3. **createList**—create files for an unsorted records for the given reference sequence and seed;
4. **sortList**—sort the records in ascending order for “values”;
5. **searchPositions**—find all candidate positions of reads by using the library of records (we also get a total number of “values” for each read and all possible and unique positions of the seed);
6. **countMatch**—for each read, we check all candidate positions in the reference sequence and report the maximum number of matching symbols and all positions where the maximum is attained.

We create several seeds using PerFSeeB tools. The output files for the ERR263486 dataset can be found in https://github.com/vtman/PerFSeeB. For reads’ length $n_r=100$, we use seeds listed in Additional file 1 (see Table 10). We use for comparison seeds $N_2$, $N_3$, $N_4$ from the table and seeds $S_1$ and $\overline{S_3}$ from Table 8 ($\overline{S_3}$ is for multiple seeds $S_1$, $S_2$, $S_4$, $S_5$, $S_6$; $S_3$ is excluded as a seed of low weight). We may see in Table 11 that the total number of reads attaining a given number of matches is the same for all seeds and 98, 99, 100 matches (or, equivalently, for the number of mismatches $n_m=2, 1, 0$). However, if we want to find reads fully matching the reference sequence at some positions, then to do this, we need 1,140,090,415 candidate positions to be checked for $S_1$, and 1,980,598,089 for five multiple seeds $\overline{S_3}$. At the same time seed $N_2$ requires only 306,980,283 candidate positions (3.7 and 6.5 times less compared to $S_1$ and $\overline{S_3}$). We also see that the average ratio of all to best candidate positions is 10 and 22 times smaller for $N_2$. And reads with two or fewer mismatches are 95% of all reads in the dataset.

**Table 10:** Seeds generated with PerFSeeB for $n_r=100$ (seeds’ weight is in the second column)

$N_2$	62	111110111001011111011100101111101110010111110111001011111011100101111101
		$\rightarrow$110010111110111
$N_3$	47	110101111000100110101111000100110101111000100110101111000100110101111000
		$\rightarrow$100110101111
$N_4$	39	1110111110010011000010110101000111011111001001100001011010100011101111
$N_5$	27	10001001011000111010000000100010010110001110100000001000100101100011101
$N_6$	23	110000101000011100000010000011000010100001110000001000001100001010000111
$N_7$	21	10001010101000000010000010100010001010101000000010000010100010001010101
$N_8$	16	100000100100100000000100000100100100000000100000100100100000000100000100
		$\rightarrow$1001

**Table 11:** For each read and its counterpart, we find the number of unique candidate positions found for given seeds and the number of positions having the maximum number of matches with the read

	$S_1$	$\overline{S_3}$	$N_2$	$N_3$	$N_4$
*Percentage of reads having at least given number of matches*					
95	96.49413	96.55955	95.88331	96.41693	96.54956
96	96.23315	96.26387	95.77562	96.20613	96.26404
97	95.81032	95.81777	95.56522	95.81777	95.81777
98	94.98875	94.98875	94.98875	94.98875	94.98875
99	92.66636	92.66636	92.66636	92.66636	92.66636
100	81.17036	81.17036	81.17036	81.17036	81.17036
*Total number of positions having the maximum number of matches*					
95	24,673	30,124	5,743	18,078	29,184
96	88,548	95,152	28,715	76,571	96,154
97	271,116	272,204	165,624	272,204	272,204
98	1,072,182	1,072,182	1,072,182	1,072,182	1,072,182
99	6,505,597	6,505,597	6,505,597	6,505,597	6,505,597
100	52,432,760	52,432,760	52,432,760	52,432,760	52,432,760
*Total number of candidate positions to be checked*					
95	2,999,321	7,694,425	62,080	534,856	5,075,260
96	6,484,344	15,560,907	183,070	1,785,944	10,215,791
97	16,738,989	35,342,085	975,884	6,106,930	23,375,078
98	49,957,296	94,777,228	6,591,836	22,966,268	66,288,831
99	206,562,418	374,668,653	42,466,387	101,753,217	268,804,889
100	1,140,090,415	1,980,598,089	306,980,283	593,471,736	1,467,713,805
*Averaged ratio of candidate positions to best positions*					
95	334.88	790.87	10.37	50.87	535.79
96	414.02	1039.40	7.57	100.63	677.69
97	493.92	1164.11	14.72	115.13	744.46
98	486.19	1087.47	22.87	137.82	704.10
99	465.90	1001.87	31.37	140.65	662.25
100	412.66	848.60	38.46	137.69	575.35

(a) Percentage of reads attaining at least a given number of matches (higher values are better). (b) The total number of positions attaining the maximum number of matches (we add up numbers for all reads, higher values are better). (c) The total number of candidate positions to be checked (smaller values are better). (d) For each read attaining the given number of matches, we find the ratio of all candidate positions to the number of best positions (averaged; smaller values are better)

Note that we may also apply new seeds as multiple seeds. For example, we may use seed $N_2$ and find all reads with at least 98 matches. Then for the remaining reads, we may use another seed. Let the total number of candidate positions to be processed for seed $N_2$ be $\beta$. If we consider seed $N_2$ followed by seed $N_3$ (denote it as $N_2 \cup N_3$), then we find all reads that have at least 97 matches, and in this case, we should process ${\approx }1.026\beta$. However, if we use $N_3$ alone, then we require $2.056\beta$ candidate positions to be processed (see numbers in Table 11). For other combinations with $N_2$ we get $1.133\beta$ for $N_2\cup N_4$, $2.099\beta$ for $N_2\cup N_5$, $2.570\beta$ for $N_2\cup N_6$, $5.029\beta$ for $N_2\cup N_7$, $7.871\beta$ for $N_2\cup N_8$. However, if we use all seeds one by one, i.e. $N_2\cup N_3\cup \ldots \cup N_8$, we get $4.434\beta$.

The proposed seeds or their combinations allow us to process a relatively small number of candidate positions and find all reads having not more than a given number of mismatches. In Table 12, we provide a number of candidate positions to be processed for each seed. We see that for seed $N_2$ applied to the real-life data, we need around 208 positions to be checked. All other seeds require significantly higher numbers of positions to be processed. The other good seeds are $S_1,\ldots ,S_6$, but they have been discussed above.

**Table 12:** The average number of candidate positions required to be checked for each read. Index “all” is used when all seeds in a group are used, and the corresponding lists of candidate positions are merged and duplicate positions removed from the final list

$B_1$	33030	$B_2$	35709	$B_3$	29874	$B_4$	28531	$B_5$	41961	$B_6$	34353
$B_7$	30830	$B_8$	27054	$B_9$	27023	$B_{10}$	33030	$B_{all}$	98503		
$M_1$	9245	$M_2^1$	11672	$M_2^2$	9657	$M_2^{all}$	16102				
$M_4^1$	13509	$M_4^2$	9742	$M_4^3$	9690	$M_4^4$	9665	$M_4^{all}$	23645		
$M_8^1$	12351	$M_8^2$	10968	$M_8^3$	10194	$M_8^4$	10456	$M_8^5$	9758	$M_8^6$	9669
$M_8^7$	9928	$M_8^8$	9524	$M_8^{all}$	29170						
$M_{16}^1$	13467	$M_{16}^2$	12357	$M_{16}^3$	11835	$M_{16}^4$	11832	$M_{16}^5$	11812	$M_{16}^6$	11247
$M_{16}^7$	10754	$M_{16}^8$	10597	$M_{16}^9$	10619	$M_{16}^{10}$	10502	$M_{16}^{11}$	10168	$M_{16}^{12}$	9762
$M_{16}^{13}$	9753	$M_{16}^{14}$	9751	$M_{16}^{15}$	9723	$M_{16}^{16}$	9724	$M_{16}^{all}$	39922		
$N_2$	208	$N_3$	423	$N_4$	1062	$N_5$	4283	$N_6$	6888	$N_7$	11529
$N_8$	22254	$R_1$	36532	$R_2$	35816	$R_3$	36393	$R_{all}$	59239	$S_3$	37046
$S_1$	825	$S_2$	674	$S_4$	504	$S_5$	624	$S_6$	706	$\overline{S_3}$	1451

Our goal is to have full-sensitivity seeds. Therefore we aim to check how the other seeds work under such requirements (full sensitivity for a given maximum number $n_m$ of mismatches). For each seed, we vary the length $n_r$ of reads and check if the seed is valid. At the same time, we may use the found size $n_r$ and design seeds according to the PerFSeeB procedure. Their weights are also found and compared to the weights of the original seeds. The weights are usually 30–40% higher compared to the weights of the original seeds. See Table 13. We may see that seeds generated directly with the extension procedure (usually $n_r < 45$) and seeds developed with the PerFSeeB approach (they are all called “best” seeds in Table 13) are valid for shorter reads: by approximately 15% for $w \le 12$, by 20% for $w=20, 22$ and by 32% for $w=39$–46.

**Table 13:** Minimum read lengths are required to achieve full sensitivity for a given number $n_m$ of mismatches

Seed	*w*	$n_m=2$	$n_m=3$	$n_m=4$	$n_m=5$	$n_m=6$	$n_m=7$
$Q_1$	4	14 (5)	16 (4)	21 (5)	24 (4)	28 (4)	30 (4)
best		**11**	**14**	**17**	**20**	**23**	**26**
$Q_2$	10	28 (13)	37 (14)	44 (12)	52	58	65
best		**22**	**29**	**37**	**44**	**52**	**58**
$P_1$	11	27 (12)	40 (15)	46 (13)	51	62	68
$P_2$		32 (16)	37 (14)	50 (14)	58	63	72
$P_3$		31 (15)	37 (14)	48 (14)	55	62	70
$P_4$		28 (13)	38 (15)	44 (12)	52	60	68
best		**23**	**31**	**40**	**46**	**56**	**62**
$D_1$	12	33 (16)	38 (15)	51 (15)	56	69	74
$D_2$		31 (15)	41 (16)	49 (14)	58	67	76
$D_3$		30 (15)	39 (15)	55 (16)	60	66	77
$D_4$		33 (16)	40 (15)	56 (17)	63 (16)	68 (17)	76
best		**25**	**33**	**43**	**50**	**59**	**66**
$S_3$	20	51 (28)	76 (34)	85 (31)	95 (26)	114 (27)	
best		**39**	**48**	**64**	**76**	**91**	
$B_1$	22	66 (38)	88 (40)	110 (44)	132 (39)	154 (38)	
$B_2$		49 (27)	68 (31)	80 (28)	94 (26)	111 (25)	
$B_3$		55 (31)	82 (38)	90 (32)	102 (28)	127 (31)	
$B_4$		51 (28)	70 (31)	88 (32)	97 (27)	112 (26)	
$B_5$		54 (30)	65 (29)	82 (29)	94 (26)	110 (25)	
$B_6$		49 (27)	76 (34)	84 (30)	97 (27)	119 (28)	
$B_7$		54 (30)	72 (32)	89 (32)	100 (27)	116 (27)	
$B_8$		58 (33)	68 (31)	91 (32)	101 (27)	117 (27)	
$B_9$		54 (30)	69 (31)	90 (32)	97 (27)	111 (25)	
$B_{10}$		55 (31)	73 (32)	88 (32)	102 (28)	113 (26)	
$R_1$		54 (30)	68 (31)	85 (31)	99 (27)	112 (26)	
$R_2$		54 (30)	74 (32)	86 (31)	98 (27)	117 (27)	
$R_3$		50 (27)	70 (31)	81 (29)	97 (27)	114 (27)	
best		**42**	**52**	**68**	**82**	**97**	
$S_1$	39	98 (61)	121 (58)	160 (69)	176 (54)		
best		**67**	**83**	**100**	**132**		
$S_6$	40	107 (68)	131 (65)	163 (72)	179 (54)		
best		**68**	**86**	**101**	**134**		
$S_5$	42	100 (62)	149 (77)	172 (76)	189 (59)		
best		**70**	**91**	**105**	**138**		
$S_2$	43	96 (60)	123 (60)	163 (72)	180 (55)		
best		**72**	**92**	**107**	**140**		
$S_4$	46	115 (74)	151 (78)	187 (82)	223 (71)		
best		**78**	**97**	**114**	**153**		

For comparison with offered seeds, we also provide numbers for the best seeds found with the PerFSeeB approach. The corresponding weights are in parentheses, and the shortest possible reads’ length for *w* and $n_m$ are in bold for each “best” row

## Discussion

The PerFSeeB approach allows us to generate long, high-weight spaced seeds for a maximum number of mismatches. The analysis of performance for the read alignment algorithm was based on the simplistic approach: a user generates a list of possible “values” and finds all candidate positions mapped with these “values” in the reference sequence. For each position of a seed to a read, we get “value” and a list of positions in the reference sequence. Thus for a read of length $n_r$ and a seed of size $n_s$, we get $(n_r-n_s+1)$ “values” and the same number of lists. If a read can be perfectly positioned in the reference sequence (100% match), then all $(n_r -n_s +1)$ lists of candidate positions should have all the same positions. Therefore we may start dealing with the intersection of all lists. Since each “value” may provide us with lists of different lengths (some “values” may have thousands of “keys”), intersecting lists may significantly reduce the number of candidate positions. If some “values” provide us with empty lists, this may help us to identify possible positions of a mismatch by analysing the location of “do not care” symbols within the seed and how lists of candidate positions vary with starting positions of the seed.

Modern data collection procedures have paired-end reads such that the distance between reads can be roughly estimated, e.g. within a thousand symbols. Therefore, one can analyse lists obtained for two reads within a pair to further reduce the number of candidate positions. This may be extremely helpful when one of the reads has insertions and deletions, as we may start working with a read not containing indels. Of course, candidate positions for relatively long reads with a small number of indels can also be found with the proposed seeds. However, proper seed design dealing with indels is still needed, so we must catch all of them. For example, seeds with relatively big chunks of “do not care” symbols inside may be processed with reads split into several parts and each part shifted by a couple of characters. If the number of reads is big, one may also try to align reads with respect to each other by comparing starting/ending chunks.

Of course, one can use the PerFSeeB approach for long reads. For example, seeds generated with the PerFSeeB approach and listed in Additional file 1 can be used for reads up to 500 symbols. However, high-weight seeds also require almost proportionally larger storage. Therefore dealing with shorter chunks of long reads may be more practical as this may also help to avoid possible insertions/deletions.

Some ideas used in the paper have already been discussed for other projects. Lossless seeds were introduced in [9] and generalised in [24]. In [36] structure of seeds for $n_m=2$ and 3 was studied in depth. In [15], the periodic spaced seeds were also discussed. However, full sensitivity seeds of maximum weight are considered for at most three mismatches. At the same time, seeds’ weights were very small, e.g. the number of 1 s in a periodic block was at most five. For this paper, we designed an optimised publicly available code to generate all possible seeds continuously. We have extensively computed all possible periodic blocks of the maximum weight for a given size *T* of a block and the number of mismatches $n_m$. For example, for $n_m=2$ and $T=70$, we found all blocks of weight 60; for $n_m=4$ and $T=50$, we get blocks of weight 24. In [41], the authors presented a method for fast computation of optimal multiple-spaced seeds of weight 11. In addition, they introduce overlap complexity, a measure which correlates with sensitivity. In the PerFSeeB approach, we design single lossless seeds (but also showed that the consequent application of several seeds reduces the number of candidates). Meanwhile, our approach does not account for a common or prohibited small substring in reference sequences. This approach was proposed in [42]. It can, in principle, be combined with the PerFSeeB approach when seeds or periodic blocks of maximum (or near maximum) weights are generated and applied to reference sequences to have the number of “keys” in library of records more evenly distributed.

## Conclusions

The PerFSeeB approach proposed in this paper is based on designing periodic blocks. When several mismatches are set, resulting spaced seeds are guaranteed to find all positions within a reference sequence. Each periodic seed consists of an integer number of periodic blocks and a “remainder” (a number of the first symbols of the block). The size of the periodic block is the difference between the read’s and seed’s lengths plus one. This relation is empirical and was observed for seeds generated by iterative extension procedure for reads of length less than 45.

Periodic blocks are found for the number mismatches $n_m$ from 2 to 9 and block’s length *T* up to 50 (or 70 for $n_m=2$); they can in be accessed at https://github.com/vtman/PerFSeeB. Those blocks can be used to generate spaced seeds required for any given length of reads. The best periodic seeds are seeds of maximum possible weight since this helps us to reduce the number of candidate positions when we try to align reads to the reference sequence. If one can generate several best seeds, we choose seeds of maximum length because this helps us reduce the number of entries to be checked in the library found for the reference sequence.

The seeds found with the PerFSeeB approach might be of lower weight for long reads (${>}200$) as we have to generate longer periodic blocks; nevertheless, they meet the requirements. Furthermore, codes used to create periodic blocks and final seeds are designed to account for SIMD instructions, can work in a multithreading environment and are publicly available at (https://github.com/vtman/PerFSeeB). While the authors did their best to minimise the number of candidate blocks to be validated, there may be trillions of them to be checked for periods *T* around 50.

The proposed approach allows us to check significantly fewer alignment positions for each read than other published seeds. This, in turn, can be exploited in sequence alignment algorithms to improve performance and ensure not to miss positions due to mismatches.

### Supplementary Information

**Additional file 1:** Maximum density of best periodic blocks as a function of block’s size and block sizes as a function of read’s length (for 2 to 9 mismatches).

## Data Availability

The codes to generate periodic blocks and seeds are publicly available at https://github.com/vtman/PerFSeeB.
