# Supplementary material for: PerFSeeB: designing long high-weight single spaced seeds for full sensitivity alignment with a given number of mismatches
Source: BMC Bioinformatics. 2023 Oct 24;24:396. doi: 10.1186/s12859-023-05517-4 (PMC10594774; doi:10.1186/s12859-023-05517-4)
Supplement: Supplementary file 1 — Additional file 1: Maximum density of best periodic blocks as a function of block’s size and block sizes as a function of read’s length (for 2 to 9 mismatches). [file 12859_2023_5517_MOESM1_ESM.pdf]

PerFSeeB: designing long high-weight single spaced seeds for full sensitivity alignment with a given number of mismatches Supplementary Material

Valeriy Titarenko and Sofya Titarenko

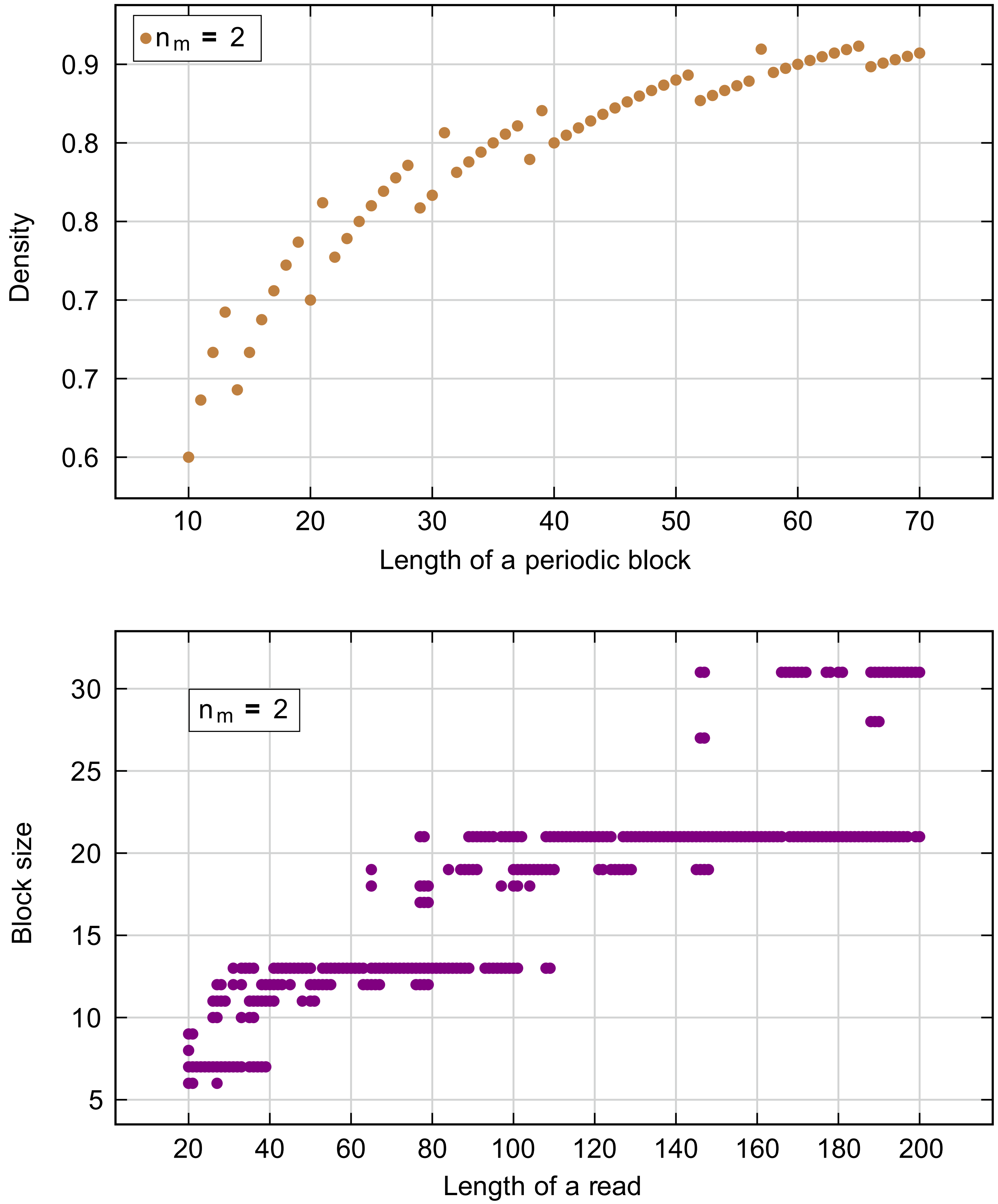

Figure S1: Maximum number of 1-elements per length of periodic blocks (top), sizes of best periodic blocks for a given length of reads (bottom), $n_m = 2$.

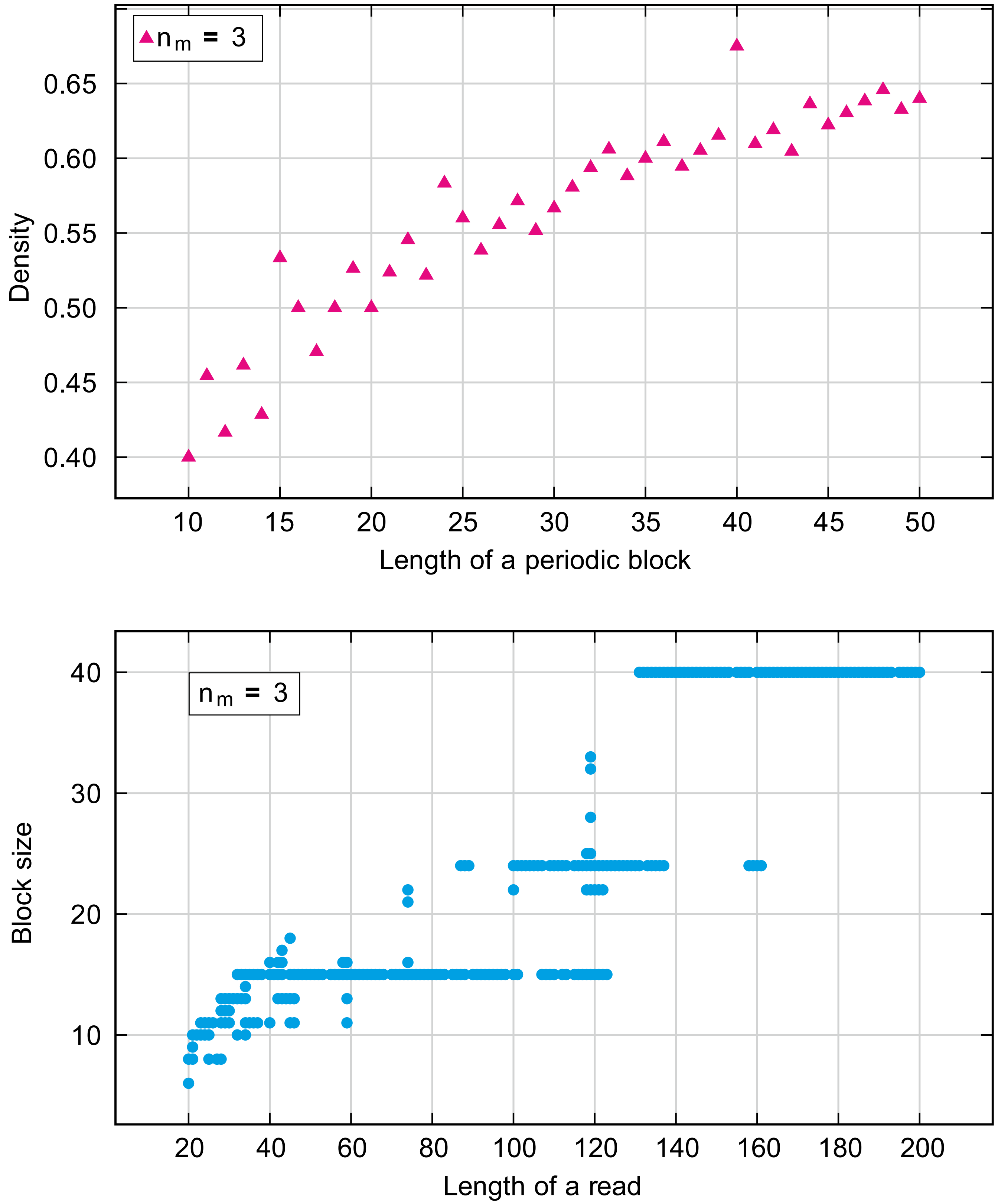

Figure S2: Maximum number of 1-elements per length of periodic blocks (top), sizes of best periodic blocks for a given length of reads (bottom), $n_m = 3$.

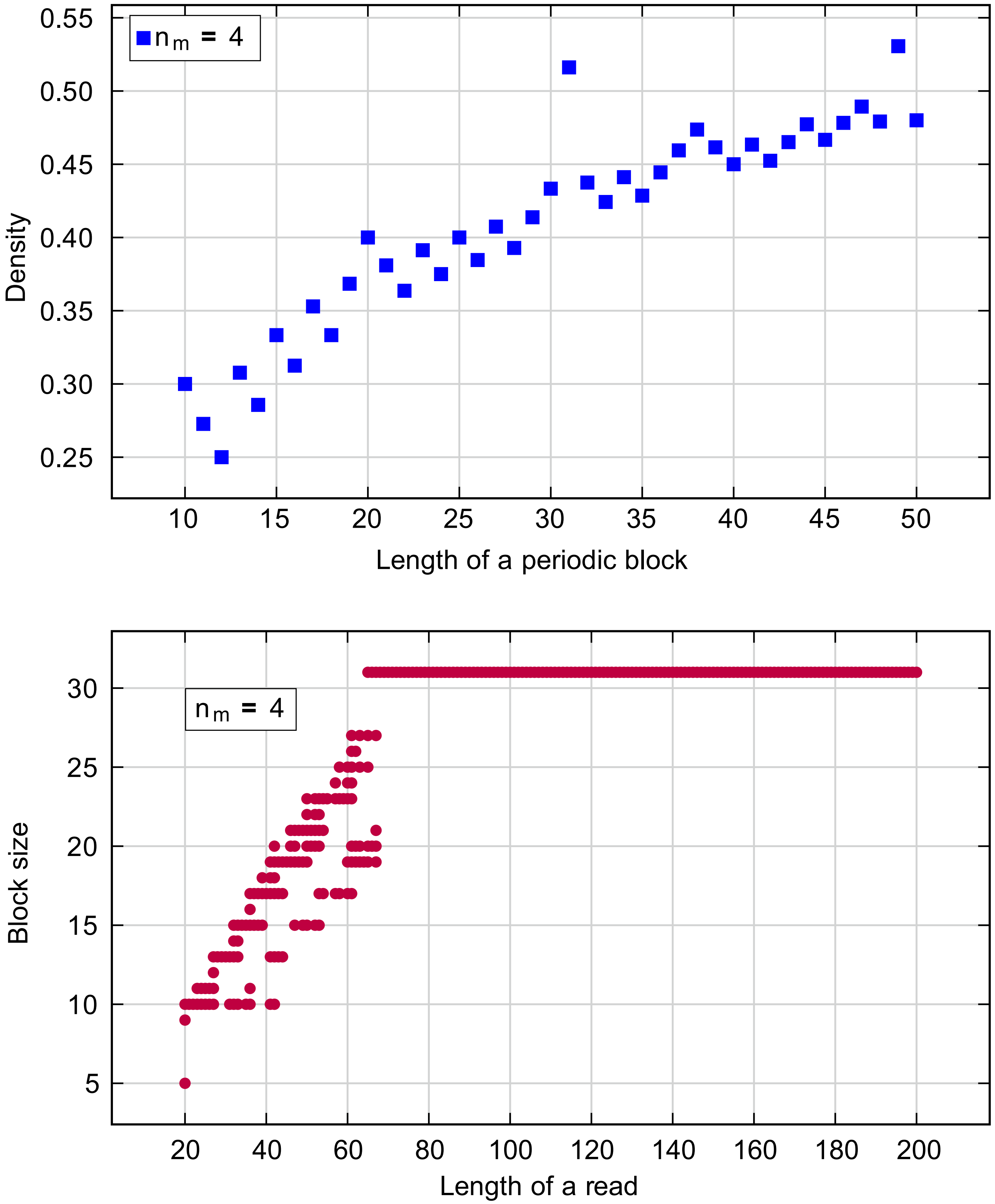

Figure S3: Maximum number of 1-elements per length of periodic blocks (top), sizes of best periodic blocks for a given length of reads (bottom), $n_m = 4$.

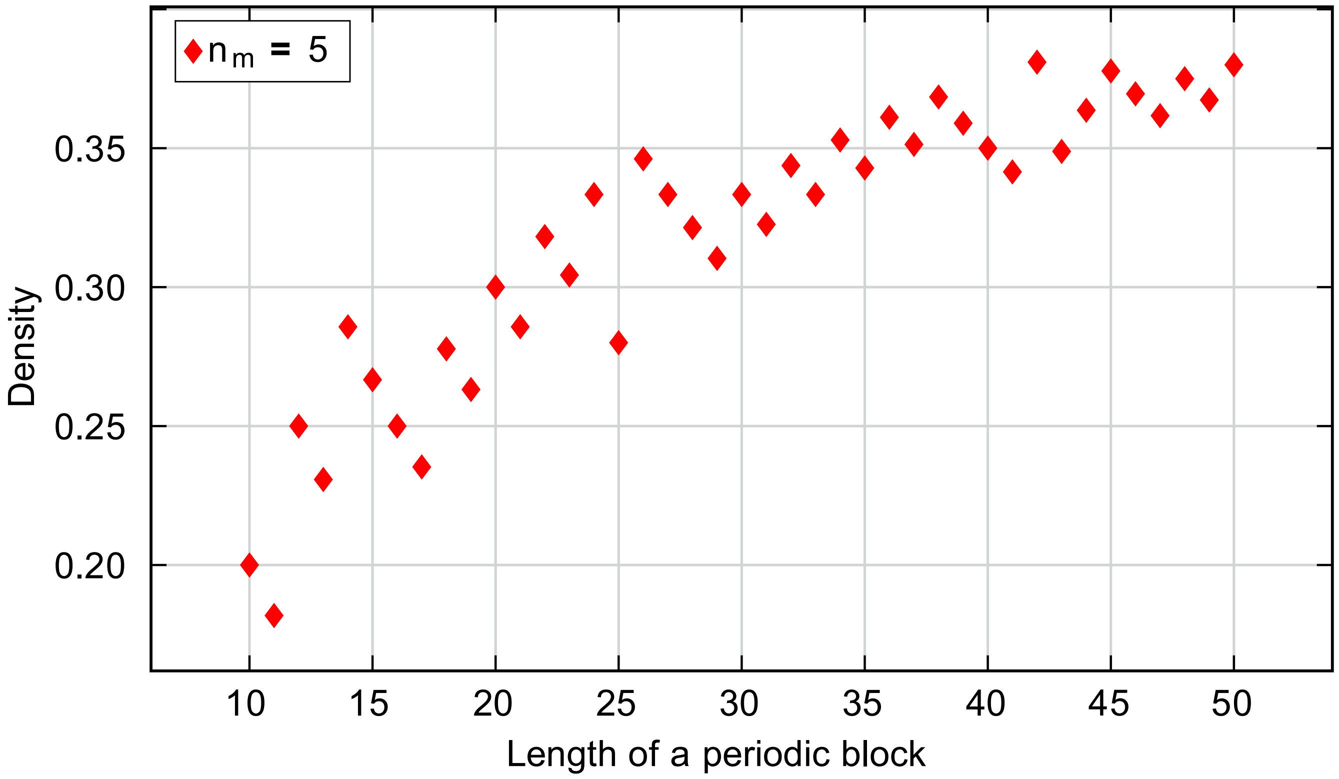

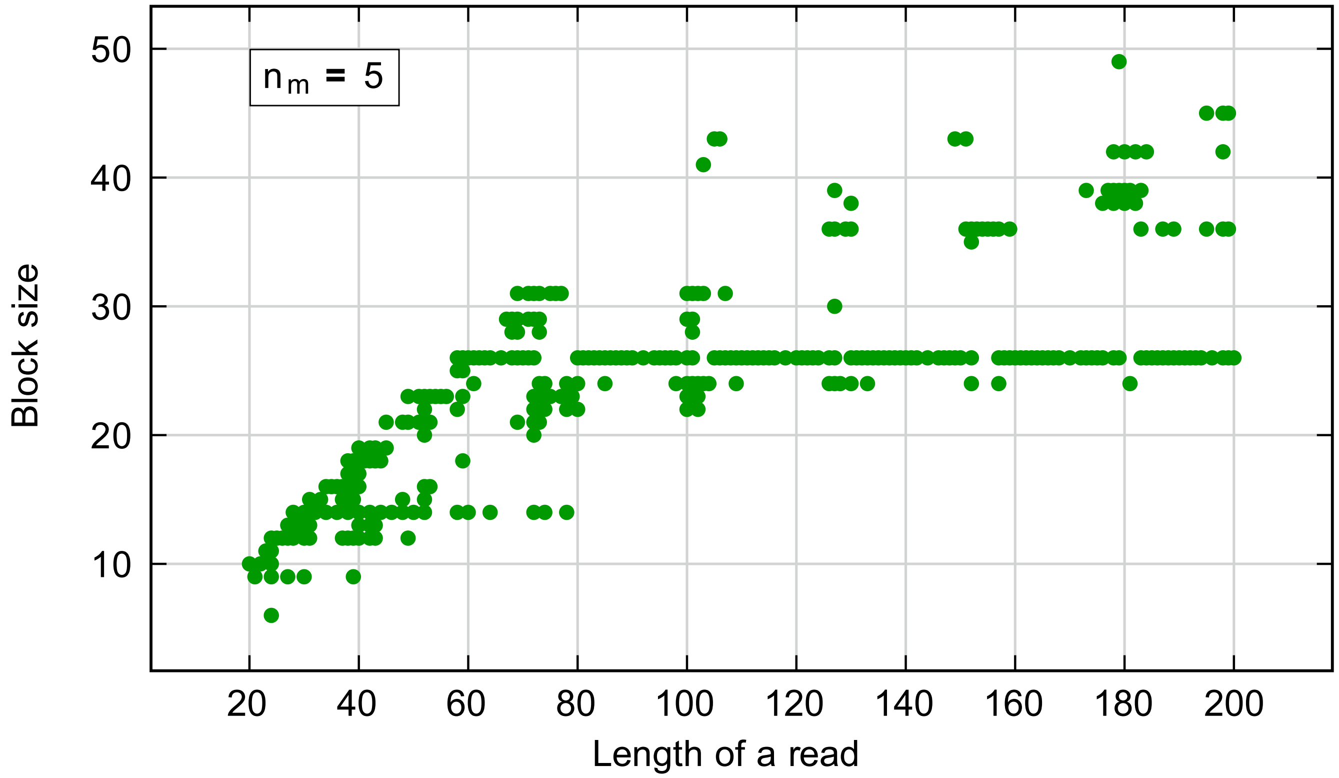

Figure S4: Maximum number of 1-elements per length of periodic blocks (top), sizes of best periodic blocks for a given length of reads (bottom), $n_m = 5$.

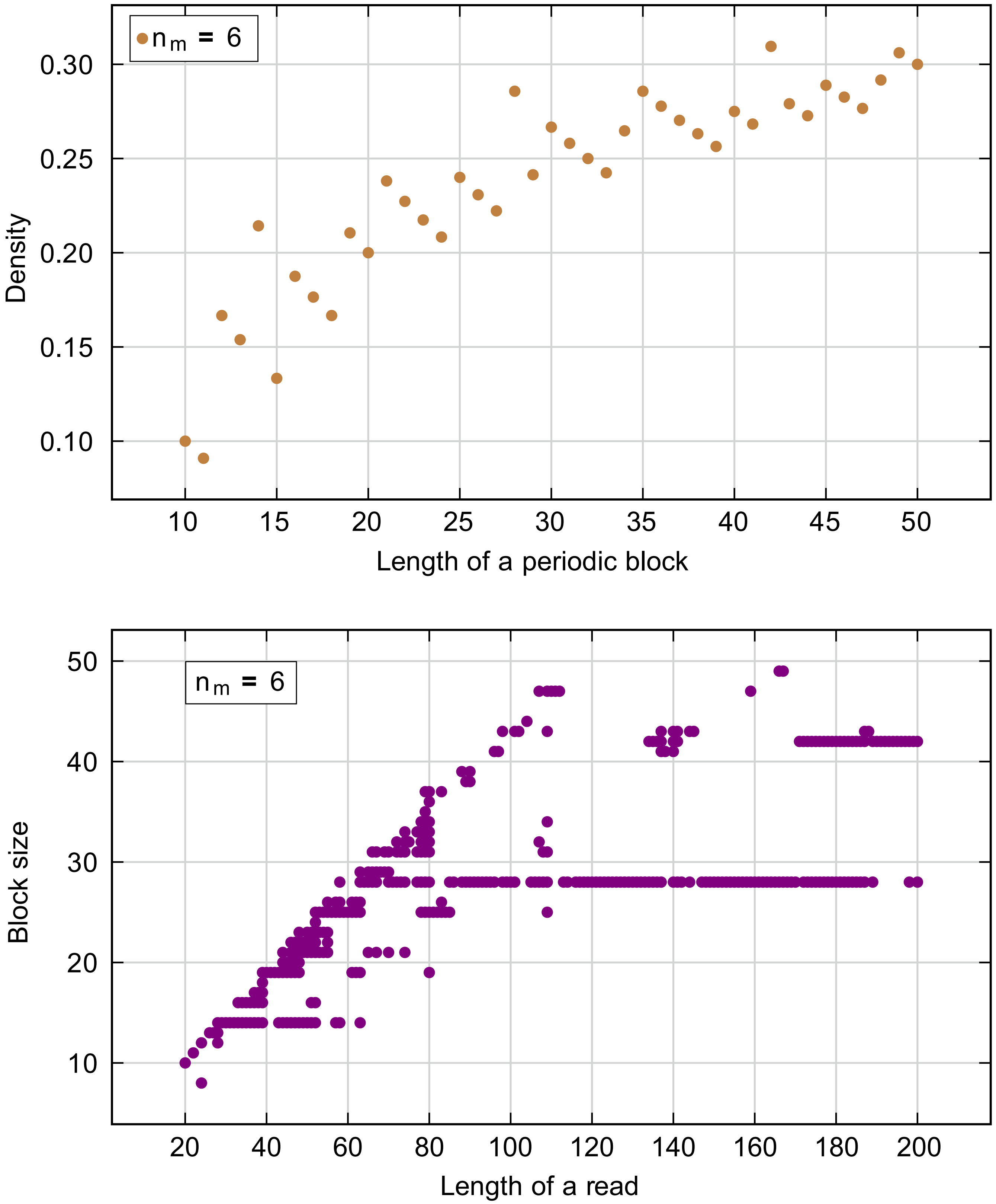

Figure S5: Maximum number of 1-elements per length of periodic blocks (top), sizes of best periodic blocks for a given length of reads (bottom), $n_m = 6$.

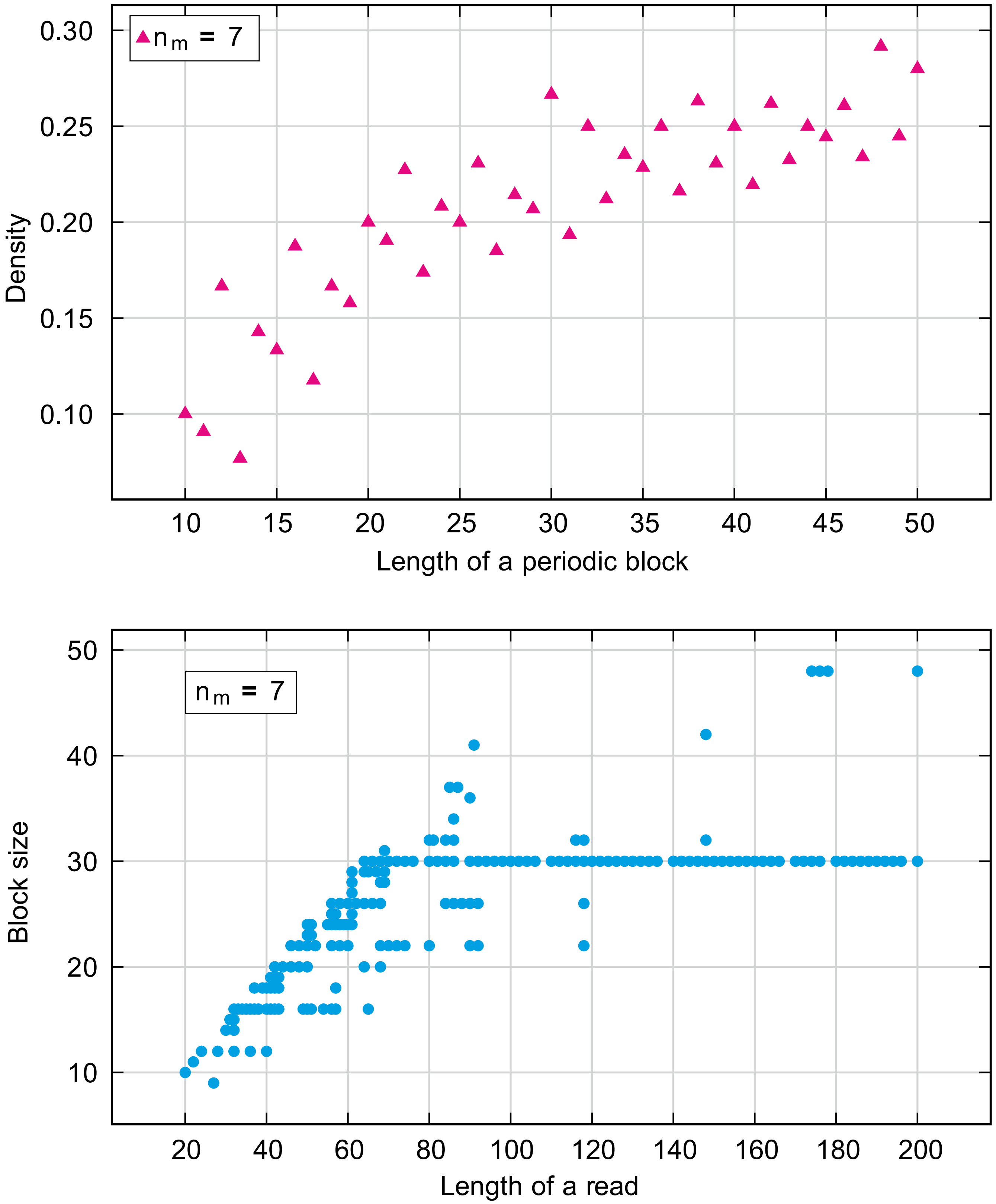

Figure S6: Maximum number of 1-elements per length of periodic blocks (top), sizes of best periodic blocks for a given length of reads (bottom), $n_m = 7$.

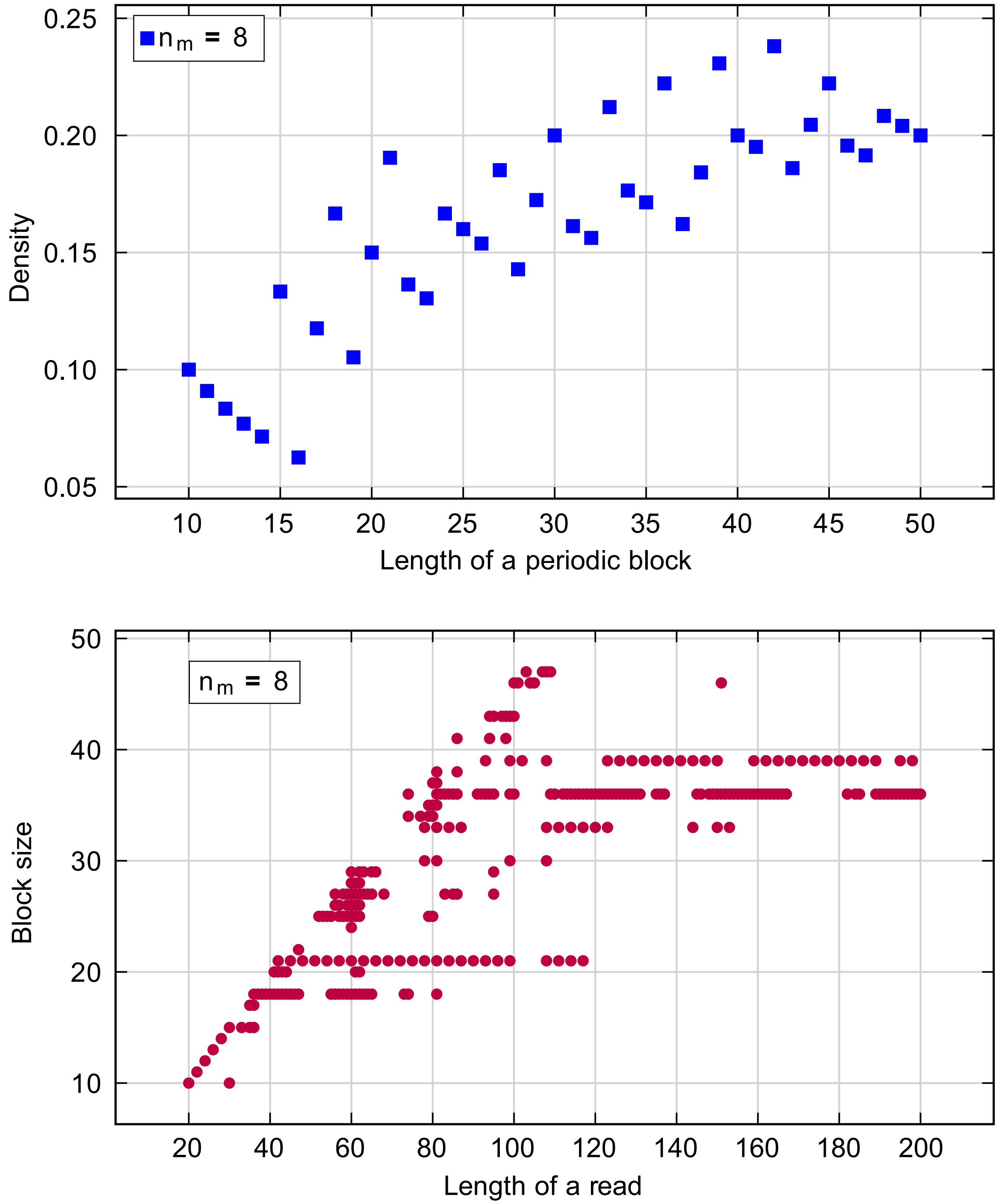

Figure S7: Maximum number of 1-elements per length of periodic blocks (top), sizes of best periodic blocks for a given length of reads (bottom), $n_m = 8$.

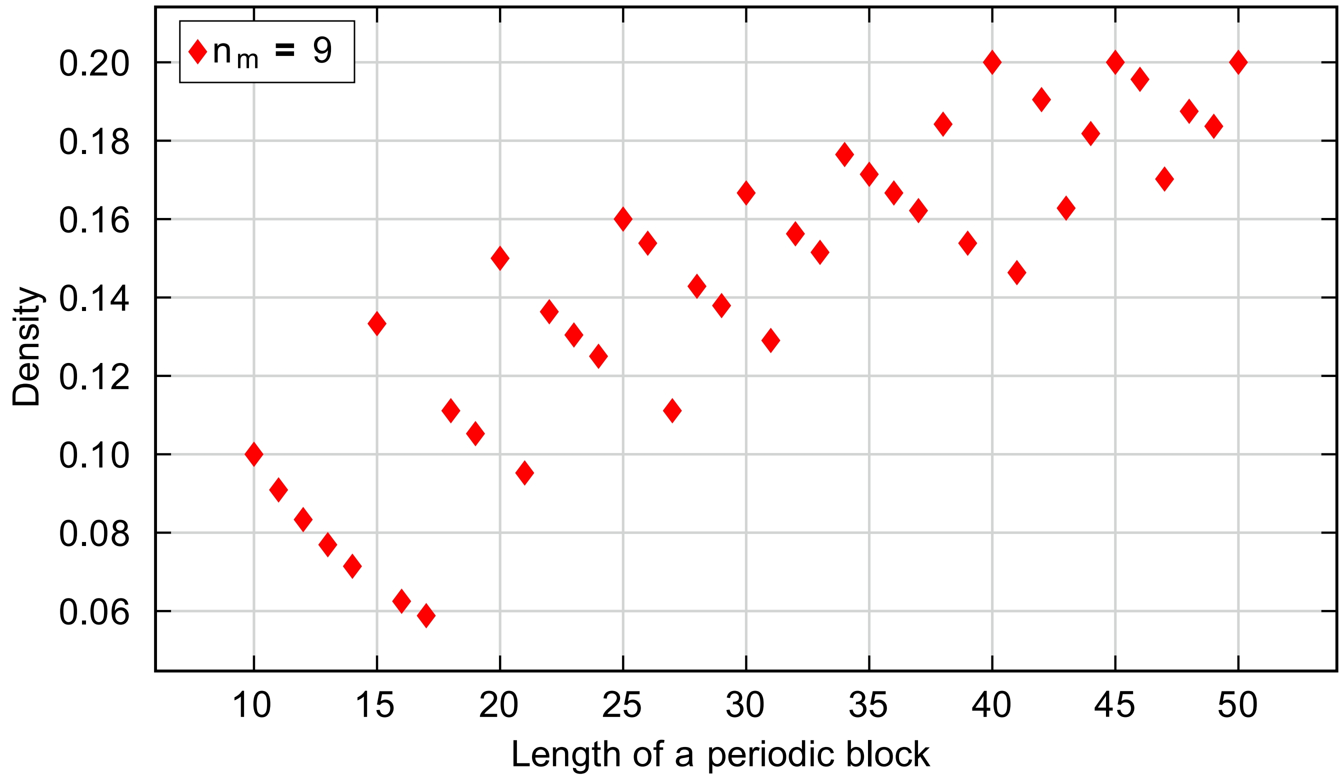

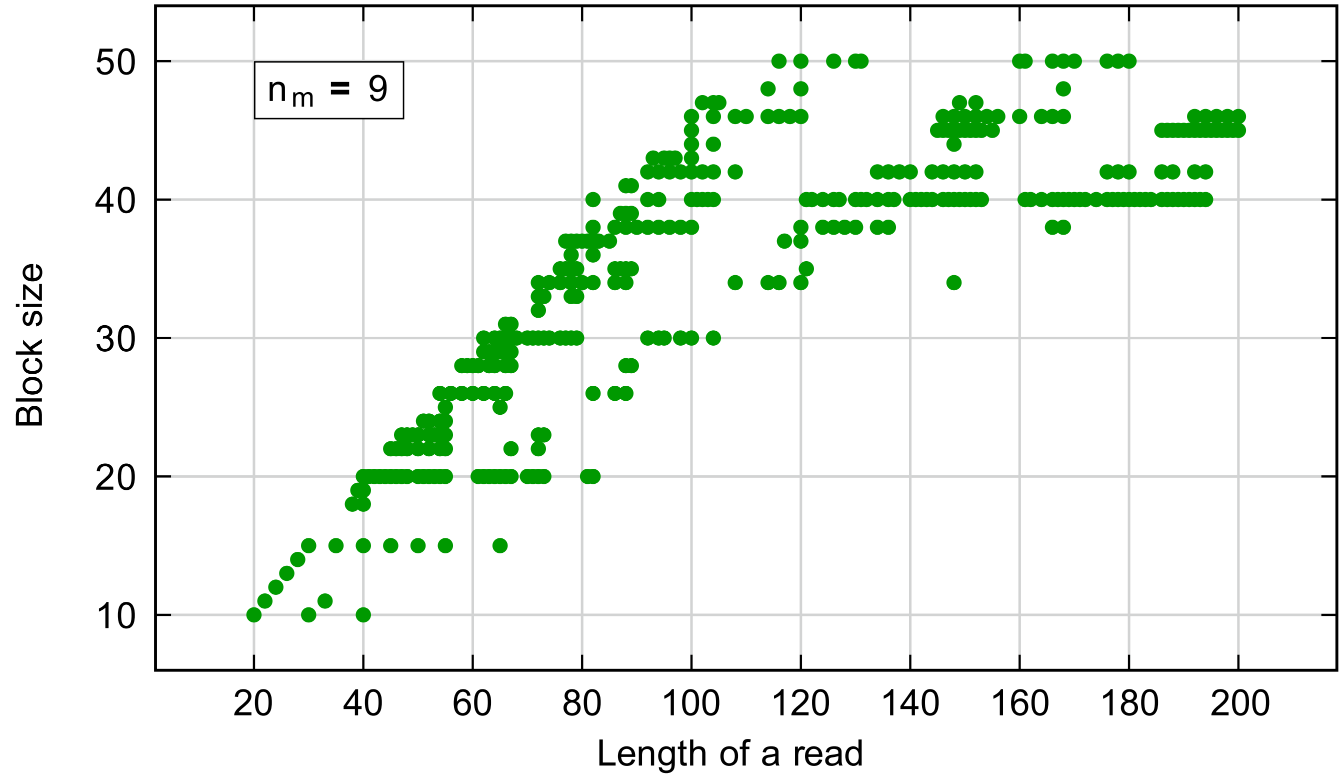

Figure S8: Maximum number of 1-elements per length of periodic blocks (top), sizes of best periodic blocks for a given length of reads (bottom), $n_m = 9$.
